# Identification of PIM1 substrates reveals a role for NDRG1 phosphorylation in prostate cancer cellular migration and invasion

**DOI:** 10.1038/s42003-020-01528-6

**Published:** 2021-01-04

**Authors:** Russell J. Ledet, Sophie E. Ruff, Yu Wang, Shruti Nayak, Jeffrey A. Schneider, Beatrix Ueberheide, Susan K. Logan, Michael J. Garabedian

**Affiliations:** 1 Departments of Biochemistry and Molecular Pharmacology, New York University School of Medicine, New York, NY 10016 USA; 2 Department of Urology, New York University School of Medicine, New York, NY 10016 USA; 3 Department of Microbiology, New York University School of Medicine, New York, NY 10016 USA; 4 Proteomics Laboratory, New York University School of Medicine, New York, NY 10016 USA

**Keywords:** Prostate cancer, Prostate cancer

## Abstract

PIM1 is a serine/threonine kinase that promotes and maintains prostate tumorigenesis. While PIM1 protein levels are elevated in prostate cancer relative to local disease, the mechanisms by which PIM1 contributes to oncogenesis have not been fully elucidated. Here, we performed a direct, unbiased chemical genetic screen to identify PIM1 substrates in prostate cancer cells. The PIM1 substrates we identified were involved in a variety of oncogenic processes, and included N-Myc Downstream-Regulated Gene 1 (NDRG1), which has reported roles in suppressing cancer cell invasion and metastasis. NDRG1 is phosphorylated by PIM1 at serine 330 (pS330), and the level of NDRG1 pS330 is associated higher grade prostate tumors. We have shown that PIM1 phosphorylation of NDRG1 at S330 reduced its stability, nuclear localization, and interaction with AR, resulting in enhanced cell migration and invasion.

## Abstract (web summary)

Through a chemical genetics screen and phospho-proteomics, Ledet and Ruff et al identify substrates of the oncogenic kinase PIM1 in prostate cancer cells. They find that NDRG1 protein is destabilized by PIM1-mediated phosphorylation to reduce its metastasis suppressor function. These findings shed light on the role of PIM1 in prostate cancer.

## Introduction

Pro-viral integration site for Moloney murine leukemia virus-1 (PIM1) is a proto-oncogene encoding a serine/threonine kinase^1,2^. PIM1 is constitutively active and does not depend upon post-translational modifications for activation^3,4^. As such, PIM1 is regulated primarily by transcription, including induction by cytokines through the JAK/STAT pathway^5^ and by hypoxia^6^. PIM1 protein levels are also regulated translationally via its 5′-UTR^7^ and by the microRNA miR-33a^8^. PIM1 has been implicated as an oncogene in both hematological malignancies, such as large B cell lymphoma^9^, and cancers of epithelial origins, such as breast and prostate cancer^10–13^. Whereas absent or weak expression of PIM1 by immunohistochemistry are observed in most benign prostate samples, moderate to strong levels of PIM1 are evident in ~50% of prostate cancers^12^.

In mouse models of prostate cancer, conditional overexpression of PIM1 in prostate epithelial cells results in prostate intraepithelial neoplasia^13^. PIM1 also cooperates with c-MYC, an established oncogene, to promote advanced prostate adenocarcinoma^10^. We and others have previously shown that PIM1 phosphorylates the androgen receptor (AR)^14^, the main driver of prostate cancer and the main target in prostate cancer therapy^15,16^. PIM1-mediated AR serine 213 phosphorylation (pS213) differentially impacts AR target gene expression and is correlated with prostate cancer recurrence^14,17^. Given that deregulation of kinases is a hallmark of cancer^18^, we hypothesized that identifying PIM1 substrates and their phosphorylation sites in prostate cancer cells could help to elucidate PIM1’s role in the disease, and help to identify cancers with active PIM1.

To identify PIM1 substrates and their phosphorylation sites in LNCaP cells, we coupled a chemical genetic screen with a peptide capture, mass spectrometry (MS)-based approach^19^. We mutated the PIM1 gatekeeper residue in the ATP binding site to accept a bulky ATP analog. By using an ATP analog labeled with a thiol group on the γ-phosphate, we were able to specifically label PIM1 substrates even in the presence of other cellular kinases^20^.

A similar approach has revealed the direct substrates of AMPK, with unexpected roles in mitosis and cytokinesis^21,22^; CDK9, with functions in transcriptional termination through phosphorylation of the 5′–3′ exonuclease XRN2;^23^ and CDK2, with a role in the DNA damage response by phosphorylation of NBS1, a necessary protein for DNA damage repair^24^. Overall, this is a rigorous approach that has revealed new substrates of kinases to yield novel mechanistic insights.

Several putative PIM1 substrates, including c-MYC, AR, and BAD, have been identified and found to be dysregulated in prostate cancer^14,17,25,26^. Still, the mechanism by which PIM1 promotes prostate cancer is not fully understood. Our study identifies new PIM1 substrates in prostate cancer cells in a direct, unbiased manner. This revealed that PIM1 substrates are involved in a variety of cellular processes, ranging from cell cycle checkpoints, to nucleic-acid metabolism, to transcriptional regulation, to cellular motility and invasion. By further exploring the PIM1-dependent phosphorylation of NDRG1, we find that PIM1-dependent phosphorylation reduces the function of NDRG1 as a metastasis suppressor.

## Results

### Identification of PIM1 substrates in prostate cancer cells

**Fig. 1:**
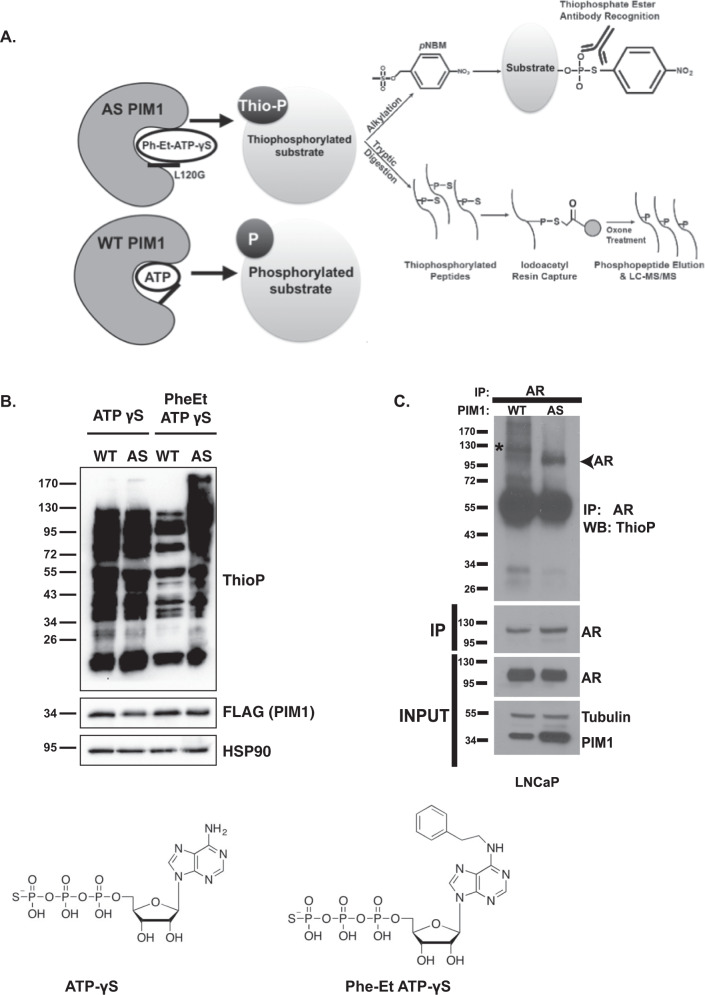
Screening strategy to identify PIM1 substrates and phosphorylation sites in prostate cancer cells. **A** Schematic of the peptide-capture technique used to identify analog-sensitive (AS) PIM1 substrates and phosphorylation sites in prostate cancer cells. AS PIM1 uses ATPγS, a bulky ATP analog, to thiophosphorylate substrates. Upper panel: thiophosphorylated substrates are alkylated by p-nitrobenzyl mesylate (PNBM) and recognized by an antibody to the thiophosphate ester moiety (ThioP). Lower panel: thiophosphorylated peptides are captured on a resin, eluted, and identified using liquid chromatography-tandem mass spectrometry (LC-MS/MS). **B** ATP analog optimization in LNCaP cells stably expressing either WT PIM1 or AS PIM1. Cells were treated with indicated ATP analog (ATP = ATPγS; PheET = N^6^-Phenylethyl ATPγS) in the presence of digitonin, a mild-permeabilizing agent. Alkylation was completed using PNBM. Whole-cell lysates were analyzed by western blot for the presence of thiophosphorylation (ThioP), PIM1 (PIM1), and HSP90 as loading control. **C** AS PIM1 thiophosphorylates endogenous AR in LNCaP cells. LNCaP-WT PIM1 and LNCaP-AS PIM1 cells were treated PhET-ATPγS as above. AR was immunoprecipitated and analyzed by western blot for the presence of thiophosphorylation (thioP) and AR. Input shows the protein levels in cells of PIM1, as well as the abundance of immunoprecipitated AR. Tubulin acts as a loading control. Western blots are representative of two independent experiments.

We conducted a chemical genetic screen to identify direct PIM1 substrates and their phosphorylation sites in LNCaP cells. We used an analog-sensitive (AS) kinase–substrate detection method^27,28^. To create the AS PIM1 kinase, we aligned the PIM1 amino-acid sequence of the 33kDA PIM1 isoform^29^ with other kinases for which AS mutants have been generated, and identified leucine 120 (L120) as the conserved gatekeeper residue (Supplementary Table 1). We mutated L120 to a smaller glycine residue (L120G) and generated pools of LNCaP cells stably expressing similar levels of either wild-type PIM1 (LNCaP-WT PIM1) or AS PIM1 (LNCaP-AS PIM1). We tested the ability of AS PIM1 to utilize the bulky N^6^-substituted ATP analog in cells by treating with digitonin, a mild-permeabilizing agent. We then lysed the cells and added *para*-nitrobenzyl mesylate (*p*NBM) to alkylate thiophosphorylated proteins, and used a thiophosphate ester-specific antibody to reveal substrates by western blot (Fig. 1A). LNCaP cells harboring the AS PIM1 were able to utilize PhET-ATPγS to phosphorylate substrates. Cells expressing WT PIM1 were much less efficient at utilizing this analog, whereas Adenosine- 5′-O-(3-thiotriphosphate) (ATPγS) is not specific for AS PIM1 (Fig. 1B). All subsequent thiophosphorylation experiments were performed with PheET-ATPγS as the ATP analog.

We have previously reported that AR is a substrate of PIM1^14^. We examined whether endogenous AR could be selectively thiophosphorylated in LNCaP-AS PIM1 as compared to LNCaP-WT PIM1 cells. Indeed, we found that AS PIM1 was able to use PhET-ATPγS to thiophosphorylate endogenous AR in LNCaP cells. However, WT PIM1 was much less effective (Fig. 1C). This indicates that the AS PIM1 retains specificity for a known substrate.

**Fig. 2:**
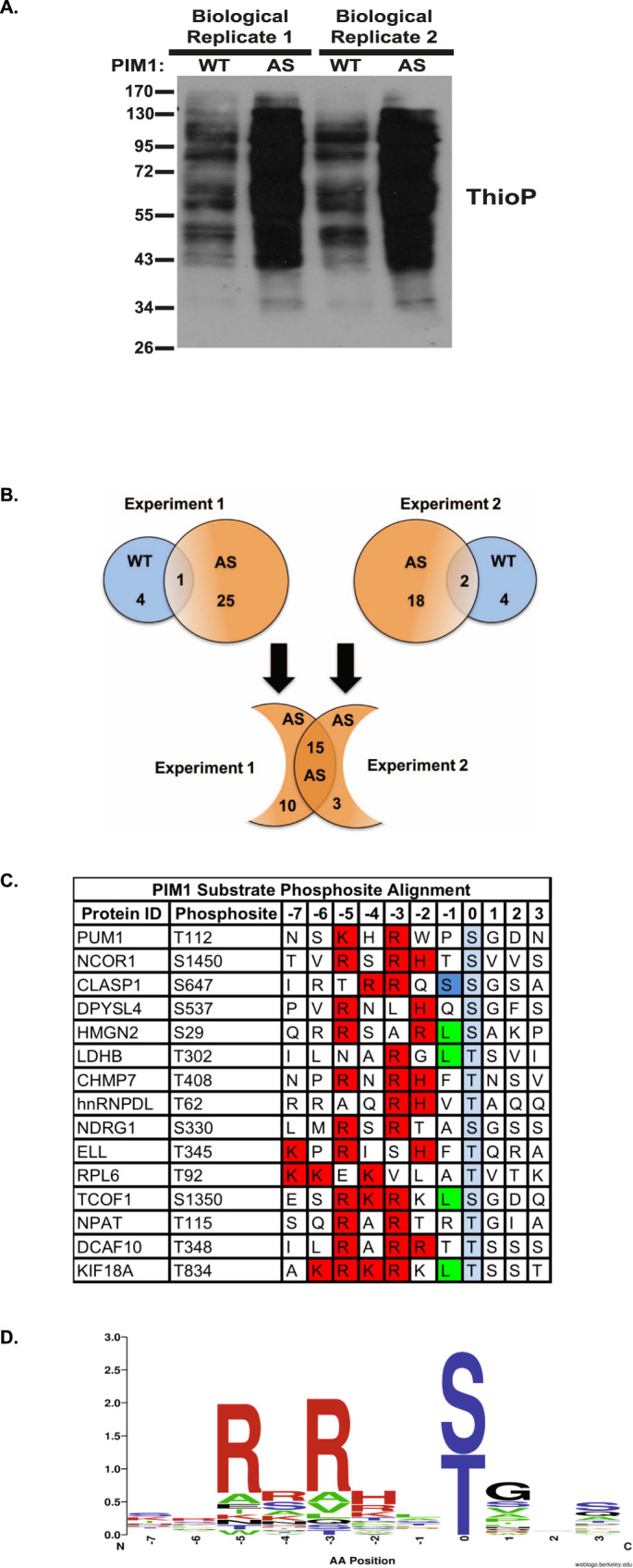
Identification of high-confidence AS PIM1 substrates in prostate cancer cells. **A** Western blot of the thiophosphate ester moiety (ThioP) from biological replicates of LNCaP-WT PIM1 and LNCaP-AS PIM1 cell lysates, 0.1% used for the phosphopeptide enrichment. **B** Proteins identified from lysates used in Fig. 2A from two independent experiments. Blue and orange circles represent proteins identified in WT PIM1 and AS PIM1-expressing LNCaP cells, respectively. **C** PIM1 substrate phosphosite alignment. Highlighted residues represent optimal, amino acids based on PIM1 consensus sequence. Red, basic residues. Green, hydrophobic residues. Blue, neutral polar residues. **D** Consensus logo generated using substrates in **C**.

To identify PIM1 substrates and their phosphorylation sites, we treated LNCaP-WT PIM1 and LNCaP-AS PIM1-expressing cells with PhET-ATPγS (Fig. 2A), and isolated thiophosphorylated tryptic peptides using iodoacetyl beads^27^. This was followed by selective release of the thiophosphorylated peptides with spontaneous hydrolysis using potassium peroxomonosulfate (OXONE), forming phosphopeptides. Liquid chromatography-tandem mass spectrometry was used to identify protein substrates and their sites of phosphorylation.

To identify and prioritize high-confidence PIM1 substrates and phosphorylation sites, we used a stringent set of criteria to analyze the MS data. Phosphopeptides found in the AS PIM1 data sets, but not in the WT PIM1 control data sets were selected. From a total of 25 protein substrates identified in two biological replicates of AS PIM1 samples (Supplementary Table 2), 15 previously unidentified PIM1 substrates were represented in both replicates (Fig. 2B–C). A PIM1 consensus motif logo was generated from the substrates found in this study (Fig. 2D). This motif conformed to the literature consensus PIM1 phosphorylation motif, R/K-X-R/K-H-X-S/T, where X is a small, neutral amino acid; and an acidic or basic residue (lysine or arginine) at the -3 and -5 positions from the phosphorylation site^30^. Therefore, these proteins likely represent high-confidence PIM1 substrates (Fig. 2C, Supplementary Table 3). We first focused on candidates, which strongly adhered to the PIM1 consensus motif, had high peptide counts, and had been previously linked to human prostate cancer. Although no previously identified substrates such as AR or c-Myc were found in our screen, we believe our screen identifies the most robust substrates of PIM1 in LNCaP cells. However, we do not think that the screen is exhaustive. For example, we previously showed that AR serine 213 is a target of PIM1. We were able to confirm this in LNCaP cells by immunoprecipitating AR from LNCaP expressing AS PIM1 and show that it can be thiophosphorylated (Fig. 1C). Given this result and that the identified substrates conform to the PIM1 consensus phosphorylation motif, we think that the paucity of known PIM1 substrates is not an issue of altered substrate specificity of the AS PIM1, but rather low abundance of the previously recognized PIM1 substrates, low levels of phosphorylation, or an inability to capture the peptides on MS^21,22^. The combination of our directed thiophosphorylation of AR from LNCaP cells expressing AS PIM1, coupled with the phosphorylation motif analysis indicating extensive alignment to the PIM1 phosphorylation consensus sequence, gives us high confidence that the substrates we identified represent bona fide PIM1 substrates, even if the screen is not saturating.

Several of the PIM1 substrates we identified are involved in the regulation of RNA and DNA metabolism. For example, PUM1, a PUF family RNA-binding protein, regulates translation of sequence-specific genes by binding their 3′-UTRs^31,32^. Substrates NCOR1 and RNA polymerase II elongation factor (ELL) have been implicated in RNA polymerase II pausing and activation, respectively^33,34^. Additional substrates identified in the screen, including CHMP7, TCOF1, NPAT, CLASP1, and KIF18A, have strong associations with mitosis, spindle formation, and microtubule depolymerization^35–40^. These connections to mitosis are intriguing, as PIM1 is known to interact with nuclear mitotic apparatus protein (NuMa) and to promote cell cycle progression during mitosis^41^. Last, NDRG1, an established metastasis suppressor involved in cell motility, and has been shown to interact with the WNT receptor LRP6 to block WNT signaling^42^. Taken together, we have identified PIM1 substrates which are involved in regulating cell cycle, nucleic-acid metabolism, cell signaling, and cell migration.

**Fig. 3:**
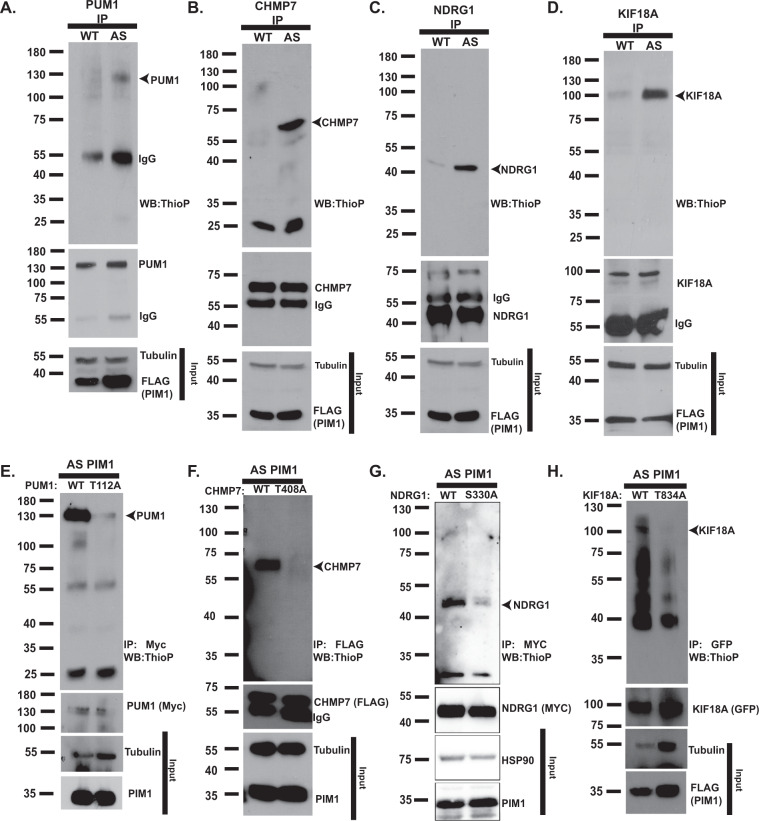
AS PIM1 directly phosphorylates endogenous high-confidence substrates in prostate cancer cells. **A**–**D** Validation of endogenous PIM1 substrates. LNCaP-AS PIM1 thiophosphorylates PUM1 **A**, CHMP7 **B**, NDRG1 **C**, and KIF18A **D**. Endogenous proteins were immunoprecipitated from LNCaP-WT PIM1 and LNCaP-AS PIM1 and analyzed by western blot for the presence of thiophosphorylation (ThioP), or immunoprecipitated PUM1, CHMP7, NDRG1, and KIF18A. **E**–**H** Phosphorylation site validation using WT and phosphorylation site mutant substrates. Substrates (Myc-PUM1, FLAG-CHMP7, FLAG-NDRG1, and GFP-6HIS-KIF18A; either wild-type (WT) or the indicated PIM1 phosphorylation site mutation) were expressed in 293 T cells with AS PIM1 and thiophosphorylation labeling completed. Substrates were immunoprecipitated using Myc, FLAG, or GFP-magnetic beads, and Western blot performed to detect thiophosphorylated, or the immunoprecipitated substrates. Western blots are representative of two independent experiments.

To validate the substrates identified in our screen, we tested endogenous thiophosphorylation of PUM1, NDRG1, CHMP7, and KIF18A in LNCaP-AS PIM1 versus LNCaP-WT PIM1 cells. All four proteins were thiophosphorylated in the presence of PhET-ATPγS by AS PIM1 to a greater extent than by WT PIM1 (Fig. 3A–D). To confirm the sites of phosphorylation, we generated serine/threonine to alanine mutants of each substrate and co-expressed them with AS PIM1 in 293 T cells. We performed the thiophosphorylation assay, including alkylation with *p*NBM, immunoprecipitated the substrate of interest, and performed Western blot using a thiophosphate ester-specific antibody. We observed reduced thiophosphorylation in the phospho-mutant substrates (Fig. 3E–H), indicating that these are the predominant sites of PIM1-mediated phosphorylation for each substrate. These data provide strong evidence that the proteins identified in our screen are PIM1 substrates in prostate cancer cells.

### NDRG1 pS330 levels vary in prostate cancer cells and tissues

**Fig. 4:**
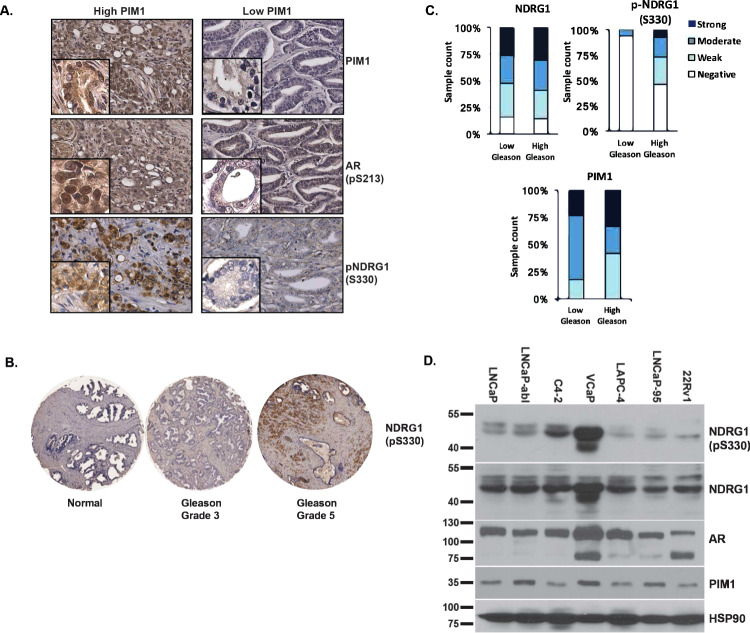
NDRG1 phosphorylation is positively associated with PIM1 expression and high Gleason grade in primary prostate cancer. **A** Immunohistochemistry (IHC) of PIM1, AR pS213, and NDRG1 pS330 in primary prostate cancer samples (*n* = 67) with low PIM1 and high PIM1 levels. **B** IHC of NDRG1 pS330 in normal prostate, low Gleason grade, and high Gleason cases. **C** IHC analysis comparing NDRG1 pS330 and total NDRG1 in low Gleason tumors versus high Gleason tumors (*n* = 76). **D** Western blot analysis of NDRG1 pS330, total NDRG1, AR, PIM1 in different prostate cancer cell lines. HSP90 was used as loading control.

We focused on characterizing the effect of PIM1-dependent serine 330 phosphorylation (pS330) of NDRG1, given that a phosphoproteomic analyses of prostate cancer tissues by Drake et al.^43^ demonstrated that levels of NDRG1 pS330 were found to be 7.7 times higher in metastatic lesions than in localized prostate cancer (Supplementary Data 1). Although several studies have implicated NDRG1 in prostate cancer^44,45^, a link between PIM1 and NDRG1 has not been established. NDRG1 is a member of the alpha/beta hydrolase superfamily, but does not possess catalytic activity^46^. It has been well documented as a potent inhibitor of tumor growth and cancer cell proliferation and an inhibitor of cell migration and invasion^47^. Its expression alters the levels and membrane localization of beta-catenin and the membrane glycoprotein KAI1 (CD82), leading to increased cell–cell adhesion^48^. In addition, overexpression of NDRG1 reduces metastasis in a rodent model of prostate cancer^49^. In contrast, prostate cancer cells with low NDRG1 expression display increased motility and invasiveness^45^, and reduced expression of NDRG1 was associated with poor overall survival in a variety of cancers, including prostate^50^. NDRG1 is transcriptionally regulated by the AR^51^. Considering this information, we examined the protein levels of NDRG1 pS330, total NDRG1, and PIM1 by immunohistochemistry using tissue microarrays containing primary prostate tumors and adjacent non-cancer tissue (Supplementary Data 2). Expression of NDRG1 pS330 and AR pS213, another PIM1 substrate, were much more prevalent in specimens with abundant PIM1 (Fig. 4A). Spearman correlation analysis revealed that NDRG1 pS330 levels in prostate cancer are associated with AR pS213 (*r* = 0.3664, *p* = 0.01) and PIM1 protein levels (*r* = 0.5038, *p* = 0.00003) (Supplementary Data 2). NDRG1 pS330 was increased in cases with high Gleason grade (Gleason 4/5) compared with tumors with low Gleason grade (Gleason 3) or non-cancer tissue (Fig. 4B, *p* = 0.044). This was observed without a significant change in total NDRG1 protein abundance (*p* = 0.583), suggesting that the change in NDRG1 pS330 was a result of increased phosphorylation by PIM1 (Fig. 4C).

We next probed a panel of prostate cancer cell lines for NDRG1 pS330, total NDRG1, and PIM1 by western blot. AR-expressing prostate cancer lines displayed varying levels of NDRG1 pS330. VCaP cells, a prostate cancer cell line with AR amplification, displayed the highest level of total NDRG1 and NDRG1 pS330, consistent with our findings that NDRG1 expression and phosphorylation are androgen-regulated (Fig. 4D; Supplementary Fig. 1). Together, these results demonstrate that NDRG1 pS330 is correlated with high-grade prostate cancers (high Gleason score), and that NDRG1 pS330 is present in multiple prostate cancer cell lines.

### PIM1-mediated phosphorylation destabilizes NDRG1

**Fig. 5:**
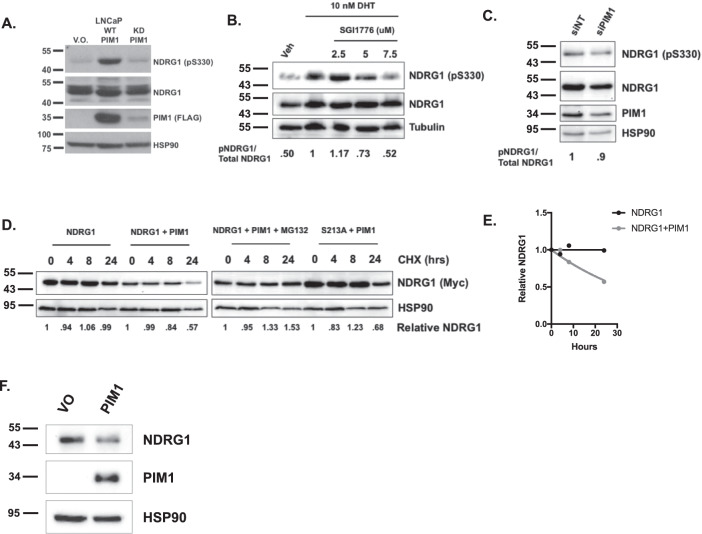
PIM1-mediated NDRG1 pS330 is androgen-dependent, and reduces its stability. **A** LNCaP cells stably expressing vector only (LNCaP V.O.), WT PIM1 (LNCaP-WT PIM1), or kinase-dead PIM1 (LNCaP KD PIM1) were steroid-starved for 48 h, and Western blot was performed for NDRG1 pS330, total NDRG1, PIM1 (FLAG), and HSP90 (loading control). **B** LNCaP and VCaP cells were steroid-starved for 48 h, treated with 10 nM DHT, along with indicated amounts of PIM1 kinase inhibitor SGI-1776 for 24 h, and western blot was performed for NDRG1 pS330, total NDRG1, and HSP90. The ratio of NDRG1 pS330/total NDRG1 was quantified using ImageJ software. **C** Under the same conditions as Fig. 2B, PIM1 was knocked down using siRNA and western blot was performed for NDRG1 pS330, total NDRG1, PIM1, and HSP90. Ratios were quantified using ImageJ software. **D** Effect of PIM1-dependent phosphorylation on NDRG1 stability. NDRG1 WT and S330A were expressed in 293 cells, with and without PIM1. Cells were treated with cycloheximide for the indicated times, lysates prepared and blotted for NDRG1 and HSP90. Additionally, WT NDRG1 + PIM1 was treated with MG-132. **E** Extrapolated half-lives of NDRG1 under the various conditions are shown. **F** PIM1 overexpression in LNCaP cells results in loss of endogenous NDRG1.

We next investigated the effect of androgens on NDRG1 pS330. As expected, in the absence of androgens, LNCaP cells overexpressing WT PIM^14^ have increased levels of NDRG1 pS330 (Fig. 5A). Treatment with the androgen dihydrotestosterone (DHT) increased NDRG1 pS330 compared to untreated cells. LNCaP cells treated with DHT and with SGI-1776, a potent and specific PIM1 inhibitor^52^, resulted in decreased NDRG1 pS330 (Fig. 5B). PIM1 knockdown by siRNA treatment also reduced NDRG1 pS330 (Fig. 5C). This indicates that NDRG1 phosphorylation at S330 is mediated by PIM1, and inhibition or knockdown of PIM kinase activity decreases phosphorylation.

To determine whether phosphorylation affects the stability of NDRG1, we examined NDRG1 stability by blocking protein synthesis with cycloheximide and tracking NDRG1 abundance over time. We co-expressed WT or phospho-mutant (S330A) NDRG1 with either empty vector or WT PIM1 in 293 cells. Compared with empty vector alone, co-expression of WT NDRG1 with WT PIM1 led to a substantial reduction in NDRG1 protein stability (Fig. 5D). NDRG1 is exceedingly stable in the absence of PIM1, with its abundance unchanged over 24 h in the presence of cycloheximide. However, the addition of PIM1 resulted in a decrease in NDRG1 abundance by 50% at 24 h (Fig. 5D). Treatment with the proteasome inhibitor MG-132 in the presence of PIM1 restored NDRG1 stability, indicating that degradation is occurring via the proteasome. By contrast, NDRG1 S330A mutant was resistant to degradation in the presence of PIM1 (Fig. 5D). Using this data, we calculated the projected half-lives for WT NDRG1 in the presence and absence of PIM1, in the presence of MG-132, and NDRG1 S330A in the presence of PIM1 (Fig. 5E, Table 1). Finally, we examined the endogenous levels of NDRG1 in LNCaP cells in the presence of PIM1 and found that PIM1 overexpression results in a reduction of NDRG1 protein (Fig. 5F). Collectively, we find that PIM1 expression leads to increased levels of NDRG1 pS330, and PIM1-dependent NDRG1 phosphorylation decreases NDRG1 protein stability.

**Table 1:** Projected half-lives for NDRG1.

Sample	NDRG1	NDRG1 + PIM1	NDRG1 + PIM1 + MG-132	S213A + PIM1
Half-life (hrs)	ind.	31.48	ind.	67.52

Although NDRG1 is stable in the absence of PIM1, the half-life is ~31 h in the presence of PIM1. The mutant S330A NDRG1 has a longer half-life of 67 h. MG-132 rescues the stability of WT NDRG1 in the presence of PIM1. *ind*. indeterminate.

### Non-phosphorylated NDRG1 is nuclear and interacts with the AR

**Fig. 6:**
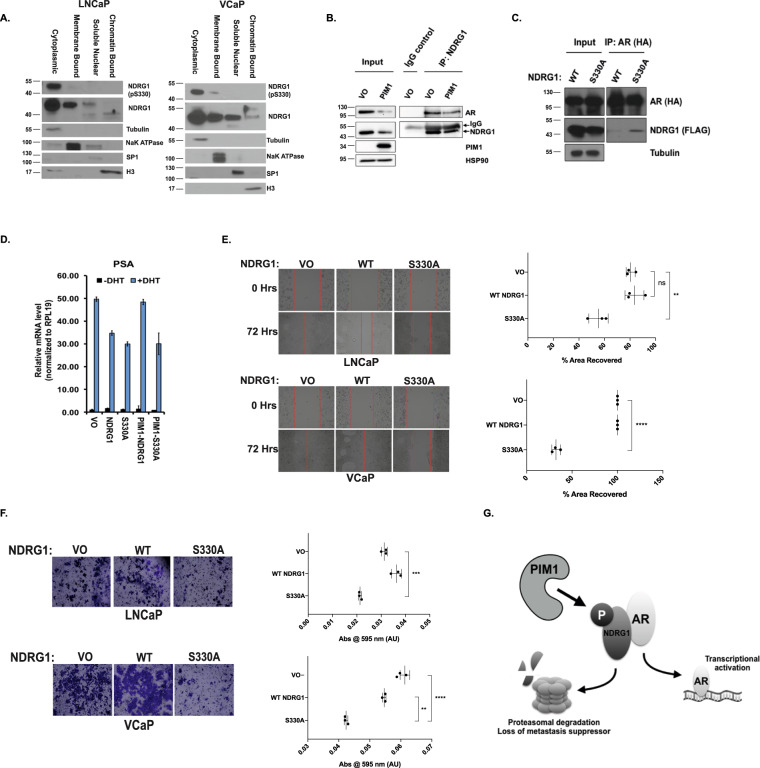
NDRG1-AR interaction is modulated by NDRG1 phosphorylation. **A** LNCaP and VCaP cells were fractionated into membrane, cytoplasmic, soluble nuclear and chromatin-bound fractions and blotted for NDRG1 pS330 and NDRG1. Markers for each compartment were also blotted for to ensure the fidelity of the fractionation. **B** Endogenous NDRG1 co-immunoprecipitates with AR in LNCaP cells. **C** AR interacts more robustly with non-phosphorylated NDRG1. 293 T cells were transfected with either NDRG1 WT or NDRG1 S330A mutant (FLAG-tagged) and WT-AR (HA-tagged). AR was immunoprecipitated using HA beads, and Western blot performed for AR (HA) and NDRG1 (FLAG). **D** LNCaP transfected with indicated genes were steroid-starved for 48 h, and subsequently treated with 10 nM DHT. mRNA levels for PSA were measured relative to RPL19 by qRT-PCR. *n* = 3 and error bars represent the standard deviation. **E**–**F** Migration and matrigel invasion assays. LNCaP-PIM1 and VCaP-PIM1-expressing cells were transfected with vector only (VO), NDRG1 WT, or NDRG1 S330A. Cell migration was determined using a scratch assay. Micrographs of the cells at 0 and 72 h are shown, and percent area remaining after 72 h was quantitated. Invasion assay through matrigel was performed in LNCaP-PIM1 and VCaP-PIM1 cells expressing the indicated NDRG1 constructs, and cells invading through matrigel were stained and shown as micrographs. The stain was extracted from the cells that migrated through the matrigel and quantitated spectrophotometrically at 595 nm wavelength. *n* = 3 independent samples for each experiment. *T* test; **p < 0.01, ***p < 0.001, ****p < 0.0001, ns not significant.  **G** Model illustrating the effect of PIM1 phosphorylation on NDRG1 and its co-repression of AR.

We next examined the subcellular distribution of NDRG1 pS330 and total NDRG1 in prostate cancer cells. PIM1 has been reported to be mainly localized to the cytoplasm^53^. LNCaP and VCaP cells were separated into cytoplasmic, membrane, soluble nuclear, and chromatin-bound fractions, and probed for NDRG1 pS330 and total NDRG1 by western blot. In both cell lines, NDRG1 pS330 was largely detected in the cytoplasmic fraction, with a small amount of NDRG1 pS330 in the membrane fraction of VCaP cells. The faster migrating NDRG1 species in the soluble nuclear fraction from LNCaP cells may represent a previously described C-terminally truncated variant of NDRG1^54^. By contrast, total NDRG1 was detected across all fractions, including the nuclear fractions (Fig. 6A). We hypothesized that phosphorylation may prevent NDRG1 from accumulating in the nucleus.

Prostate cancer is highly dependent on AR transcriptional activity^55^. For this reason, we examined whether NDRG1 interacts with AR and affect its transcriptional activity. We immunoprecipitated endogenous NDRG1 from LNCaP cells, with or without PIM1 overexpression (Fig. 6B). We were able to observe co-immunoprecipitation of AR with NDRG1. In addition, consistent with our previous results, the overexpression of PIM1 reduced the abundance of NDRG1 protein, resulting in a reduction in immunoprecipitated NDRG1 and AR. Next, to determine whether S330 phosphorylation affects the interaction between NDRG1 and AR, we over-expressed WT or S330A NDRG1 in 293 T cells (Fig. 6C). We immunoprecipitated AR and observed that it interacts more robustly with NDRG1 S330A than WT NDRG1. This suggests that non-phosphorylated form of NDRG1 is more capable of interacting with AR potentially through alterations in its abundance or localization.

Because we observed an interaction between AR and NDRG1, we tested whether NDRG1 affected AR transcriptional activity. We transfected LNCaP cells with NDRG1 or the non-phosphorylatable NDRG1 S330A, in the absence and presence of PIM1, and assayed the expression of AR target genes PSA and NKX3.1. Expression of WT NDRG1, and to a greater extent S330A, decreased androgen-dependent expression of PSA and NKX3.1 (Fig. 6D, Supplementary Fig. 2A). Co-expression of PIM1 with NDRG1 rescued the PSA and NKX3.1 expression in the presence of WT NDRG1. This suggests that NDRG1 negatively regulates AR activity, and that PIM1-dependent NDRG1 pS330 reverses this effect. Collectively, this data suggest that non-phosphorylated NDRG1 interacts with and represses AR transcriptional activity, and upon phosphorylation at S330 by PIM1, this interaction and co-repression is reduced.

### NDRG1 S330A suppresses cell invasion and migration

Multiple reports have illustrated the role of NDRG1 as a metastasis suppressor^42,45,56^. For example, overexpression of NDRG1 in a mouse xenograft model reduced lung and liver metastasis^57^. NDRG1 regulates the expression of the membrane protein KAI1 (CD82), which interacts with adjacent cells via the membrane protein gp-Fγ to inhibit metastasis^57^. Moreover, NDRG1 expression in HepG2 cells leads to increased membrane-localized beta-catenin, which promotes a less-migratory phenotype and greater cell–cell contact^45^. In light of these findings, and given the previously demonstrated ability of PIM1 to promote cell migration and invasion^58,59^, we investigated the effect of NDRG1 phosphorylation on the migration and invasion of prostate cancer cells. To examine migration, we exogenously expressed WT NDRG1 or NDRG1 S330A in PIM1 overexpressing LNCaP and VCaP cells (Supplementary Fig. 2C), and used a scratch assay to monitor migration over 72 h. In LNCaP-WT PIM1-expressing cells, there was an 80% scratch area recovery after 72 h (80.55 ± 2.065, *n* = 3). WT NDRG1 overexpression was unable to suppress this cell migration (83.55 ± 4.299, *n* = 3, *p* = 0.5628), but in cells expressing NDRG1 S330A, there was only a 55% recovery (55.31 ± 4.394, *n* = 3, *p* = 0.0065) (Fig. 6E). Likewise, in VCaP cells expressing WT PIM1 alone or with WT NDRG1, 100% scratch closure was observed in all replicates after 72 h, and the area recovery was only 32% in cells expressing NDRG1 S330A (32.29 ± 2.7, *n* = 3, *p* < 0.0001) (Fig. 6E). Knockdown of NDRG1 by siRNA in LNCaP cells also increased cell migration (Supplementary Fig. 2B). Similar results were observed in a matrigel invasion assay. In LNCaP cells expressing WT PIM1 alone (0.03133 ± 0.0006667, *n* = 3, measured as absorbance at 570 nm), no decrease in invasion was noted upon overexpressing WT NDRG1 (0.03633 ± 0.001202, *n* = 3). However, there was a significant decrease in invasion upon overexpression of S330A (0.02133 ± 0.0003333, *n* = 3, *p* = 0.0002). In VCaP cells, WT NDRG1 expression resulted in a small decrease in invasion (0.05467 ± 0.0003333, *n* = 3, *p* = 0.0021) when compared with WT PIM1 alone (0.06133 ± 0.0008819). However, a much greater reduction in invasion was seen with overexpression of S330A (0.04233 ± 0.0003333, *n* = 3, *p* < 0.0001) (Fig. 6F). KAI1 expression is also increased with overexpression of NDRG1 S330A in LNCaP cells (Supplementary Fig. 2D). These results suggest that phosphorylation of NDRG1 reduces its function as a suppressor of migration and invasion, and that blocking PIM1-dependent NDRG1 phosphorylation could restore its metastasis suppressor function.

## Discussion

We used an unbiased method to identify direct PIM1 substrates in prostate cancer cells. We identified 25 previously unknown PIM1 substrates and their phosphorylation sites. We demonstrated that the specificity of the AS PIM1 was retained, as AS PIM1 is still able to thiophosphorylate the known PIM1 substrate, AR^14^. Of the 25 substrates that were present in AS PIM1 and not WT PIM1, 15 were identified in two biological replicates, and the phosphorylation sites conform to the PIM1 consensus sequence. We validated four of these targets using alkylation and detection by western blot. Our screen, however, was not comprehensive, and did not identify other previously identified PIM1 substrates, such as c-MYC. This may reflect low abundance of the phosphorylated substrates in LNCaP cells, or may be due to the fact that not all tryptic peptides are equally eluted from iodoacetyl beads or equally detectable by MS. In addition, our approach represents a direct method for identifying PIM1 substrates in prostate cancer cells. Therefore, these substrates may be the most representative of PIM1 activity in prostate cancer. In fact, PIM1 has overlapping substrate specificity with AKT protein kinases: for example, both have been shown to phosphorylate the pro-apoptotic protein BAD^60,61^. However, data from in vivo studies using knock-out mice indicate that AKT and PIM kinases are mechanistically distinct in the control of hematopoietic cell proliferation^62^. This reinforces the notion that identification of direct PIM1 targets is key to elucidating its function in a given cell type.

We have found that PIM1 substrates are involved in multiple physiological processes. Network enrichment analysis of all 25 substrates using Metascape^63^ [http://metascape.org] revealed functional classes including chromosome segregation, pre-RNA complex, nucleotide catabolic processes, transcriptional regulation, and infectious disease (Supplementary Fig. 3A). This is consistent with studies of PIM1’s effect on cell cycle, transcription, and cell motility. In addition, a majority of the substrates fall into a protein interaction network that suggests PIM1 phosphorylation may coordinate protein complexes to impact cellular function (Supplementary Fig. 3B). Further studies will be required to validate the interaction network and determine whether these associations are phosphorylation-dependent.

Among the newly identified PIM1 substrates, we focused on NDRG1. NDRG1 was shown to be as an inhibitor of metastasis. Its expression alters the expression and membrane localization of beta-catenin and the membrane glycoprotein KAI1 (CD82), leading to increased cell–cell adhesion^57^. NDRG1 is transcriptionally regulated by AR, which is also phosphorylated by PIM1. In addition, Drake et al.^43^ reported that the level of NDRG1 pS330 was higher in metastatic castration-resistant prostate cancer lesions than in treatment-naive, localized prostate cancer. This was the only phosphorylation site identified in our screen that was increased in metastatic compared with localized prostate cancer (Supplementary Data 1). Immunohistochemical analysis of prostate cancer tissue microarrays showed a significant increase in NDRG1 pS330 in malignant compared to benign tissues. This correlates with the low expression of PIM1 in benign tissues and moderate to high PIM1 expression in prostate cancer^12^. PIM1-dependent phosphorylation of NDRG1 appears to also reduce its stability, with the NDRG1 S330A mutant showing an increased half-life in the presence of PIM1. Previous studies have suggested that NDRG1 protein stability is reduced by the fusion of SUMO2 to NDRG1^64^. Whether phosphorylation of NDRG1 results in NDRG1 sumolyation to control protein stability remains to be addressed.

The migratory and invasive potential of cancer cells are key mediators of metastasis^18^. PIM1 expression has been correlated with lymph node metastasis and poor prognosis in patients with lung adenocarcinoma and squamous cell carcinoma^65^. We were interested in how PIM1 kinase activity could promote metastasis via one of the substrates we identified. Indeed, the non-phosphorylatable NDRG1 S330A suppressed migration and invasion of LNCaP and VCaP cells. We have shown that PIM1 phosphorylation of NDRG1 reduces its stability, reduces its nuclear localization, reduces its interaction with AR, and therefore relieves AR co-repression. This suggests that PIM1-mediated phosphorylation of NDRG1 inhibits its ability to exert metastasis-suppressive functions in prostate cancer. Our results establish an understanding of how phosphorylation inhibits the function of NDRG1 in suppressing migration and invasion. Based on these findings, we propose a model whereby PIM1 is, in part, imposing its oncogenic function by phosphorylating NDRG1 to reduce its metastasis suppressor activity (Fig. 6G). Thus, identifying direct substrates has allowed for the characterization of the mechanism by which PIM1 mediates an oncogenic phenotype—namely cell migration and invasion—in the context of prostate cancer. We anticipate that the characterization of additional PIM1 substrates will lend further mechanistic insights into the oncogenic properties imparted by PIM1 in prostate cancer.

## Methods

### Cell culture and reagents

Cell lines used in this study were purchased from ATCC, with the exception of LNCaP-95^66^, LNCaP-abl^67^, and LAPC-4 cells, which were gifts from Dr. J. Luo (Johns Hopkins University), Dr. Z. Culig (Innsbruck Medical University, Austria), and Dr. R. Reiter (University of California, Los Angeles) respectively. C4–2 cells were purchased from Characterized Cell Line Core Facility at MD Anderson Cancer Center (Houston, TX). Both LNCaP-95 and LNCaP-abl cells have been authenticated by short tandem repeat profiling. 293 T and VCaP cells were cultured in DMEM supplemented with 10% fetal bovine serum, 1% penicillin/streptomycin. LNCaP, 22Rv1, and C4–2 cells were cultured in RPMI (containing phenol red and l-glutamine) supplemented with 10% fetal bovine serum, 1% penicillin/streptomycin. LNCaP-abl and LNCaP-95 cells were cultured in RPMI (without phenol red) supplemented with 10% charcoal-stripped fetal bovine serum, 1% penicillin/streptomycin, 1% l-glutamine. LAPC-4 cells were cultured in IMDM supplemented with 10% fetal bovine serum, 1% penicillin/streptomycin. All cell lines were regularly assessed for mycoplasma contamination.

### Plasmids, primers, and antibodies

Constructs utilized are as follows: pCDNA 3.1 (+)-AR-FLAG^14^, NDRG1 (HG14119-CF, Sino Biologicals), CHMP7 (HG14273-CF, Sino Biologicals), PUM1 (RC201219, Origene), KIF18A (Addgene plasmid # 23002), pCDNA 3.1 (+)-WT PIM1^14^, pCDNA 3.1 (+)-KD (K67M)^14^, pLB(N)CX-WT PIM1^14^, pLB(N)CX-KD PIM1^14^. pCDNA 3.1 (+)-AS (L120G) PIM1 and pLB(N)CX-AS PIM1 (L120G) was generated using site-directed mutagenesis. WT, KD, and AS PIM1 plasmids contain the 33 kDa PIM1-S isoform. Primers are as follows for mutagenesis: PIM1 L120G (5′-gctcgggcctctccccgatcaggacgaaac-3′), NDRG1 S330A (5′-cgctggaaccagcggctgtgcggga-3′), PUM1 T112A (5′-gcaaacatcgatggcctgctggggataacattcat-3′), KIF18A T834A (5′-actgtttgatgtagaacttgctaatttccgtttccttttggcag-3′), CHMP7 T408A (5′-cacgctgttggcaaaatgcctattgcgggggt-3′). Resulting products were transformed in competent DH5α cells (Thermofisher). Site-directed mutagenesis was performed using the QuikChange Lightning Multi Site-Directed Mutagenesis kit (210515, Agilent) according to manufacturer’s instructions. All plasmids were sequenced to confirm the mutations and to ensure no additional charges in the sequence were introduced. The antibodies used were as follows: PIM1 (sc-13513; Santa Cruz Biotechnology), NDRG1 (HPA006881, Sigma), NDRG1 pS330 (ab124713, Abcam), AR (sc-7305, Santa Cruz Biotechnology), HSP90 (610418, BD Biosciences), FLAG (F3165, Sigma), α-Tubulin (ab7291, Abcam), CHMP7 (HPA036119, Sigma), Thiophosphate ester antibody [51–8] (ab92570, Abcam), KIF18A (C-19, Santa Cruz Biotechnology), PUM1 (ab92545, Abcam), NaK^+^ ATPase (EP1845Y, Abcam), SP1 (PA5-29165, Thermofisher), Histone H3 (ab1791, Abcam).

### Quantitative RT-PCR

Total RNA was extracted using the RNA-easy mini kit (Qiagen Inc., Valencia, CA) and reverse transcribed using the Verso cDNA synthesis kit (ThermoFisher Scientific). As previously described, data were analyzed using the ΔΔCT method and normalized to RPL19^14^. Primers can be found in Supplementary Table 4.

### Transfection and transduction

Transient transfections were carried out utilizing Lipofectamine 2000 (Invitrogen) according to the manufacturer’s protocol. Viral transductions were carried out in 293 T cells (4.5 × 10^6^ per 10 cm plate) utilizing 5 μg target retroviral construct, pLB(N)CX-ORF (where ORF represents the gene of interest), 2 μg VSV-G (Addgene plasmid # 8454), and 3 μg pGag-POL (Addgene plasmid # 35614) utilizing Lipofectamine (Invitrogen). Target cells, plated at 2 × 10^6^ per 6 cm plate, were treated with viral supernatant, supplemented with polybrene (8 μg/mL, H9268, Sigma dissolved in dH_2_O) for 4 h, and media replaced. Viral supernatant treatment completed twice over a 48 h period, and pools selected using Blasticidin S (A1113903, Thermofisher) (8 μg/mL). Selected pools were maintained in Blasticidin S (1 μg/mL).

### Thiophosphorylation of PIM1 substrates

Thiophosphorylation experiments were carried out as described with minor modifications^27^. In brief, 2.0 × 10^5^ HEK293T cells were transiently transfected with 1 μg of WT or AS (L120G) PIM1 and 1 μg of candidate substrate in poly-d-lysine coated six-well plates using Lipofectamine 2000 according to manufacturer’s protocol. After 48 h cells are washed 2× PBS, and treated with 150 μL thiophosphorylation buffer (20 mM HEPES pH 7.3, 100 mM KOAc, 5 mM NaOAc, 2 mM MgOAc_2_, 1 mM EGTA, 10 mM MgCl2, 0.5 mM DTT, 5 mM creatine phosphate (#2380; Calbiochem), 57 mg/ml creatine kinase (#2384; Calbiochem), 30 µg/ml digitonin (D141, Sigma), 5 mM GTP (G8877, Sigma), 0.1 mM ATP (A1852, Sigma), 0.1 mM ATPγS, or N^6^-(2-Phenylethyl)adenosine-5′-O-(3-thiotriphosphate) (PheET-ATPγS) (#A060 and P026, respectively, Biolog, Germany), 0.45 mM AMP (A1752, Sigma), 10 mM calyculin A (#990; Cell Signaling), and 1× protease inhibitor cocktail (#5871, Cell Signaling) for 20 min with gentle rotation at room temperature. 150 μL of a modified 2× radioimmunoprecipitation (RIPA) buffer (100 mM Tris [pH 8.0, 300 mM NaCl, 2% NP-40, 0.2% sodium dodecyl sulfate (SDS), and 20 mM ethylenediaminetetraacetic acid (EDTA)) containing 5 mM *p*NBM (alkylating agent) was added and incubated at room temperature for 60 min. Following alkylation, lysates were collected and cell debris removed by centrifugation at 14,000 RPM for 10 min at 4 °C. Protein lysates were suspended in 6× SDS loading buffer and boiled for 5 min. Proteins were resolved by SDS-polyacrylamide gel electrophoresis (PAGE) and analyzed with the designated antibodies.

For mass spectrometric analysis, 4.0 × 10^6^ LNCAP cells stably expressing either WT PIM1 or AS (L120G) PIM1 were plated in poly-d-lysine coated 100 mm plates and allowed to attach overnight. A total of 10 plates per replicate were utilized for mass spectrometric analysis. For each plate, 500 μL of thiophosphorylation buffer was used. Protein samples (3 mg) were spiked with 1 μg of thiophosphorylated myelin basic protein as enrichment control and the samples were lysed and digested into peptides as described in Hertz et al.^27^ with the following modifications. Briefly, 3 mL of cell lysate samples were resuspended in 9 mL of denaturation buffer (8 M urea solution in 100 mM ammonium bicarbonate, 2 mM EDTA, 1 M TCEP). Samples were reduced at 55 °C for 1 h. Post reduction samples were cooled to room temperature and diluted using 50 mM ammonium bicarbonate so that the final urea concentration was 2 M to facilitate enzymatic digestion. To ensure the samples remained in a reduced state, additional TCEP was added to a final concentration of 10 mM. Samples were digested overnight at 37 °C using sequencing grade modified trypsin. Samples were acidified using trifluoroacetic acid (TFA) to a final concentration of 0.2% to stop digestion. Peptides were loaded onto a C18 Sep-Pak cartridge (Waters) equilibrated with 0.1% TFA and desalted by washing with 9 mL of 0.1% TFA. Peptides were eluted stepwise with 40% followed by 80% acetonitrile in 0.5% acetic acid and the eluatates were combined and concentrated using a SpeedVac. The desalted peptide mixture was subjected to covalent capture of thiophosphorylated peptides as described in ref. ^27^.

Using the auto sampler of an Easy nLC1000 (Thermo Scientific), an aliquot of each enriched sample was injected onto a trap column (Acclaim® PepMap 100 pre-column, 75 μm × 2 cm, 3 μm, 100 Å, Thermo Scientific) coupled to an analytical column (EASY-Spray PepMap column, 50 m × 75 μm ID, 2 μm, 100 Å, Thermo Scientific). Peptides were gradient eluted into a Q Exactive (Thermo Scientific) mass spectrometer using a 60 min gradient from 2 to 31% solvent B (90% acetonitrile, 0.5% acetic acid), followed by 10 min from 31 to 40% solvent B and 10 min from 40 to 100% solvent B. The Q Exactive mass spectrometer acquired high-resolution full MS spectra with a resolution of 70,000 (at *m/z* 400), an AGC target of 1e6 with maximum ion time of 120 ms, and a scan range of 400–1500 *m/z*. Following each full MS, 20 data-dependent high-resolution HCD MS/MS spectra were acquired using a resolution of 17,500 (at *m/z* 400), AGC target of 5e4 with maximum ion time of 120 ms, one microscan, 2 *m/z* isolation window, fixed first mass of 150 *m/z*, normalized collision energy of 27, and dynamic exclusion of 30 s. The MS/MS spectra were searched against the Uniprot human reference database using Byonic^68^ (Protein Metrics) with the following parameters: oxidation of methionine (M), deamidation of asparagine (N) and glutamine (Q), phosphorylation of serine (S), threonine (T), and tyrosine (Y) were selected as variable modifications. Both precursor and fragment mass tolerances were set to 10 ppm. All identified peptides were filtered using a Byonic score of >200. And a false-discovery rate of 0.01 was applied for protein level identification.

### Immunoprecipitation of candidate substrates

For immunoprecipitation after thiophosphorylation and alkylation referred to in “Thiophosphorylation of PIM1 Substrates” (Fig. 3), 40 μl out of 500 μl lysate was retained to evaluate expression. The remaining lysate was incubated with 25 μL of FLAG agarose beads (M2, Sigma, F2426) pre-washed 3× in bead wash buffer (50 mM Tris HCl pH 7.5, 100 mM NaCl, 5 mM EDTA, and 0.4% Triton-X), and incubated for 2 h at 4 °C with end-over-end rotation. In order to recover immunoprecipitated protein, beads were washed 5× with bead wash buffer containing protease inhibitors. To elute proteins, beads were incubated with 100 μL TBS containing FLAG peptide for 1 h at 4 °C with end-over-end rotation. Beads were pelleted and the 6× SDS loading buffer added to eluted proteins in the supernatant and boiled for 5 min. Proteins were resolved by SDS-PAGE and analyzed by western blotting with the appropriate antibodies. All uncropped western blots are located in Supplementary Fig. 4.

### NDRG1 protein stability assay

In all, 293 cells were transfected with indicated constructs using Lipofectamine 2000 (Invitrogen) according to manufacturer’s protocol. Eighteen hours post transfection, 50 μg/mL of cycloheximide (C7698, Sigma) was added to cells for the indicated times. Cells were lysed in 1× RIPA supplemented with 1× complete protease inhibitors, and 10 μM calyculin A. Protein lysates were resuspended in 6× SDS loading buffer and boiled for 5 min. Proteins resolved by SDS-PAGE and analyzed with NDRG1 and PIM1 antibodies. For LNCaP cells expressing vector only or PIM1^14^, cells were treated with cycloheximide for the indicated times, lysates prepared and NDRG1 and PIM1 abundance determined. For MG-132 experiments, cells were treated with 10 μM MG-132 for the duration of the treatment in the presence of cycloheximide.

### Migration and matrigel invasion assay

For the migration assay, LNCaP and VCaP cells were plated in six-well plates, at 500,000 cells per well and transfected with indicated constructs using Lipofectamine 2000 according to manufacturer’s instructions. After 48 h, cells were plated into two-well silicone insert with a defined cell-free gap, according to manufacturer’s instructions (#81176, Ibidi USA). LNCaP cells were plated at 350,000/mL, and VCaP cells were plated at 850,000/mL. After an overnight incubation, the inserts were removed, and images captured after 72 h. Images were obtained at ×5 magnification utilizing an EVOS FLc. The area recovered was measured by ImageJ software in arbitrary units, and normalized to the starting area from day zero. Error bars represent standard deviation for three distinct samples. For the invasion assay, matrigel-coated chambers were rehydrated with serum-free DMEM. In all, 100,000 LNCaP or VCaP cells were added to the upper chamber in 500 µL of media. In the lower chamber 500 µL of complete media was added to serve as the chemoattractant. After 48 h, cells that did not invade matrigel were cleared away using a cotton swab, and remaining cells were stained with 0.05% crystal violet dye. For quantification, chambers were incubated for 2 mins in 150 µL of 10% acetic acid in dH_2_O to solubilize the dye taken up by the cells and quantified spectrophotometrically at 570 nm. Error bars represent standard deviation for three distinct samples. All statistical tests for migration and invasion assays were unpaired, two-tailed, parametric *t* tests.

### Human prostate cancer tissue microarrays, immunohistochemistry, and scoring

Human prostate tissue microarrays are from US Biomax, Inc. (Rockville, MD) (cat # PR803a and PR807b). Tissues slides were deparaffinized and rehydrated in xylenes and a series of graded ethanol. Antigen retrieval was performed with 0.01 M citrate buffer at pH 6.0 for 20 min at 95 °C. Slides were allowed to cool for another 30 min, followed by sequential rinsing in phosphate-buffered saline with 0.01% Triton-X (PBS-T). Endogenous peroxidase activity was quenched by incubation in PBS-T containing 3% hydrogen peroxide. Each incubation step was carried out at room temperature and was followed by three sequential washes in PBS-T. Sections were incubated in 5% normal goat serum in RT for 1 h before incubation with rabbit polyclonal NDRG1 pS330, total NDRG1, PIM1, AR pS213, and total AR (441) antibodies, overnight at 4 °C. The next day, slides were washed with PBS-T three times and were incubated with biotinylated secondary antibody for 1 h, peroxidase-labeled streptavidin (Vectastain system, Vector Laboratories) for 1 h, and diaminobenzidine substrate for peroxidase-based immunohistochemistry (Cardassian DAB Chromogen, Biocare Medical). Slides were counter-stained with hematoxylin (Vector Laboratories) and dehydrated before mounting. The intensity of immunohistochemistry staining was scored as negative (0), weak (1), moderate (2), or strong staining (3). Comparison between NDRG1 p330 score and AR pS213 score in high versus low PIM1-expressing cases was analyzed by Student’s *t* test. Relationship between NDRG1 p330 in high and low Gleason grade cases was analyzed by Student’s *t* test. The association among NDRG1 p330, total NDRG1, and PIM1 were calculated using Spearman correlation coefficient analysis.

### Statistics and reproducibility

Statistical testing was completed using Prism 7 for Mac OS X, Version 7.0e. Samples were randomly allocated into experimental groups. Samples were randomly allocated into experimental groups, and no data were excluded from the analysis. Experiments were repeated 2–3 times and had high reproducibility. All replicates used for statistical testing represent biological replicates.

### Reporting summary

Further information on research design is available in the Nature Research Reporting Summary linked to this article.

## Supplementary information

Supplementary Information

Description of Additional Supplementary Files

Supplementary Data 1

Supplementary Data 2

Supplementary Data 3

Reporting Summary

## Data Availability

All data generated or analyzed during this study are included in this published article (and its supplementary information files). Mass spec data are available under MassIVE ID: MSV000085028.
