# Supplementary material for: Identification of PIM1 substrates reveals a role for NDRG1 phosphorylation in prostate cancer cellular migration and invasion
Source: Commun Biol. 2021 Jan 4;4:36. doi: 10.1038/s42003-020-01528-6 (PMC7782530; doi:10.1038/s42003-020-01528-6)
Supplement: Supplementary file 1 — Supplementary Information [file 42003_2020_1528_MOESM1_ESM.pdf]

Supplemenatary Figures

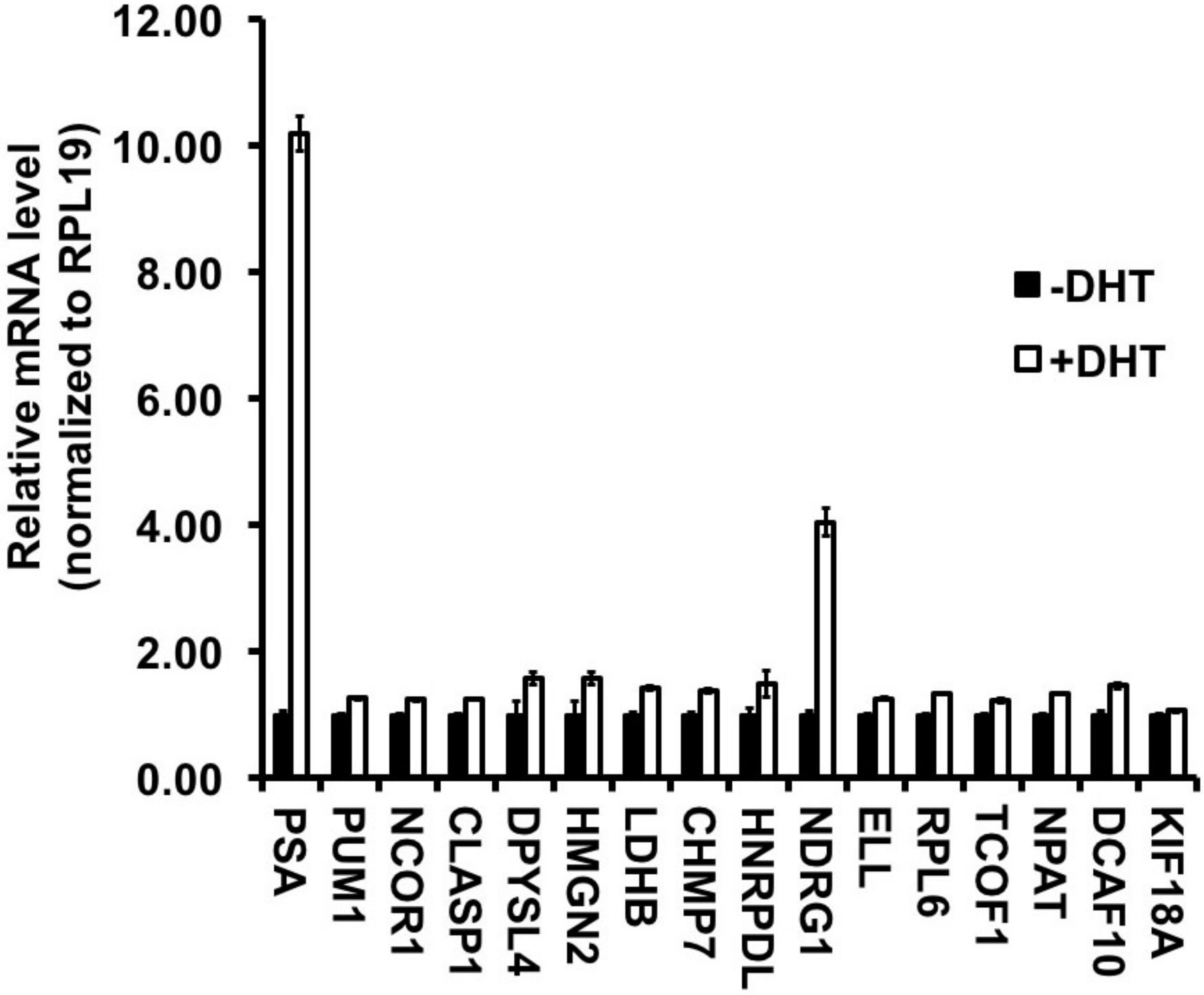

Supplementary Figure 1. NDRG1 expression is stimulated by androgen-treatment in LNCaP cells. LNCaP cells were steroid-starved for 48 h, and treated with 10 nM DHT for 24 h. mRNA levels for PSA, and PIM1 substrates relative to RPL19 were analyzed by qRT-PCR.

Supplementary Figure 2. Supplemental Figures Associated with Figure 6

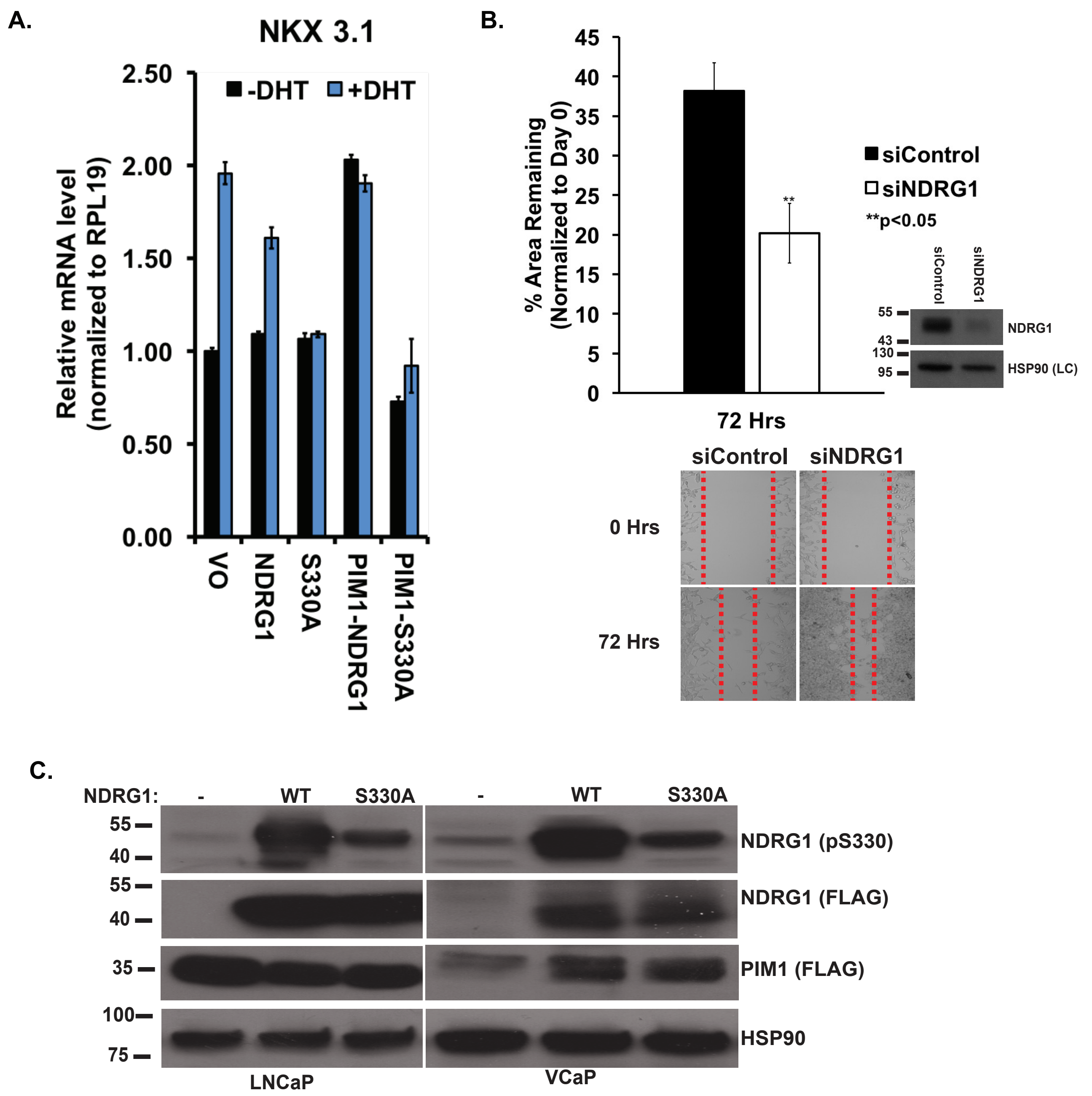

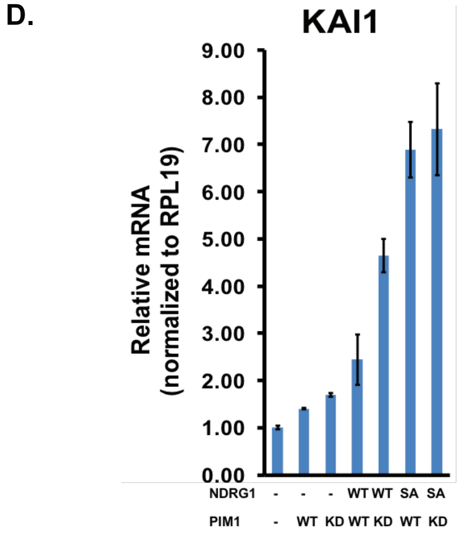

Supplementary Figure 2. Supplementary figures associated with Figure 6. A) LNCaP cells were transfected with the empty vector, NDRG1 or NDRG1 S330A, without or with PIM1, steroid-starved for 48 h, and treated with 10 nM DHT for 24 h. mRNA expression of Nkx3.1 was measured by qRT-PCR relative to RPL19. B) LNCaP cells were transfected with either non-targeting siRNA (siControl) or NDRG1 siRNA (siNDRG1). After 48 h, cells were plated for migration assay, and area remaining quantified by ImageJ analysis. Inset illustrates NDRG1 knockdown by siRNA by Western blot. C) Western blot analysis from LNCaP and VCaP cells of exogenously expressing empty vector (-), wild type NDRG1 (WT), or NDRG1 S330A (SA) in the presence of WT PIM1. HSP90 was used as loading control. D) (Pertaining to Figure 6D-E) LNCaP cells were transfected with either NDRG1 WT or NDRG1 S330A and/or WT PIM1 or kinase dead (KD) PIM1, and KAI1 gene expression was measured by qRT-PCR relative to RPL19.

Supplementary Figure 3. Metascape Analysis of PIM1 Substrates

A.

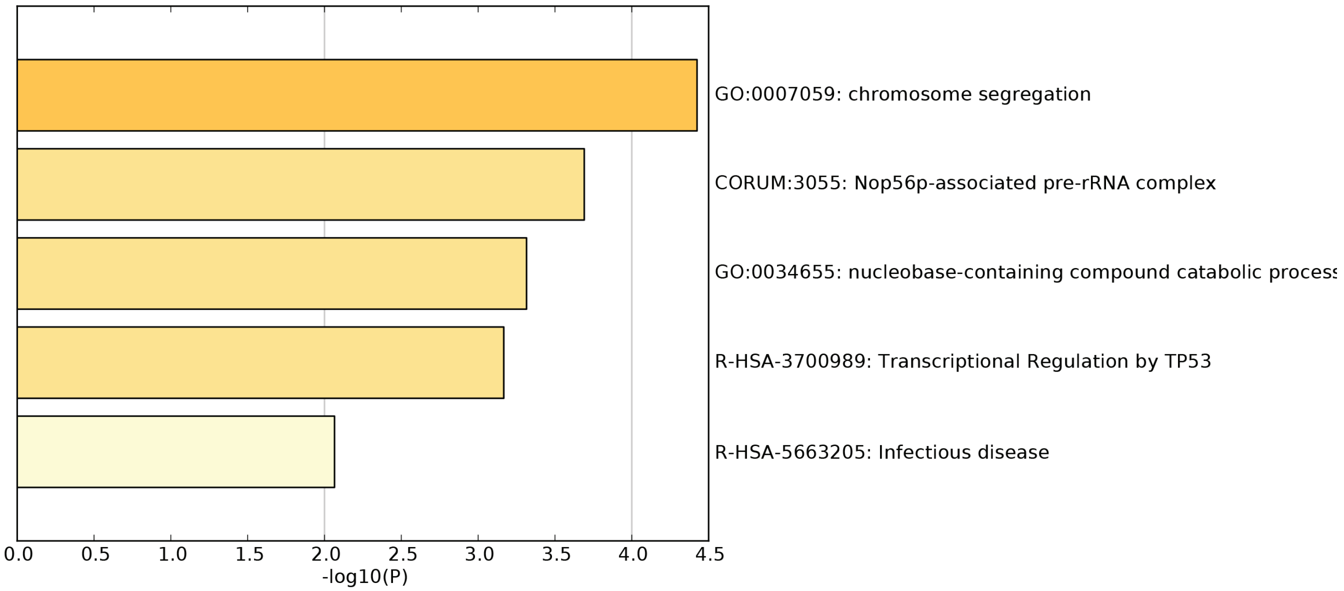

B.

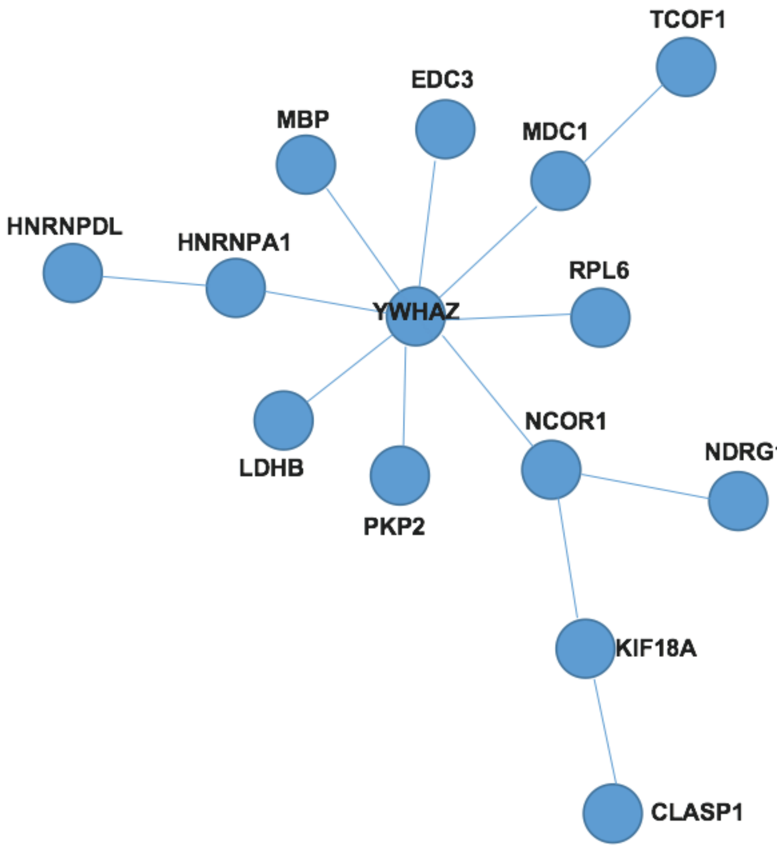

Supplementary Figure 3. Metascape analysis of gene ontology and protein-protein interactions of PIM1 substrates. A) Heatmap of enriched terms of the 25 PIM1 substrates from LNCaP cells colored by p-values. B) Protein-protein interaction network of the PIM1 substrates from LNCaP cells.

Supplementary Figure 4

Supplementary Figure 4. Full Western blots for main figures.

Figure 1

ThioP

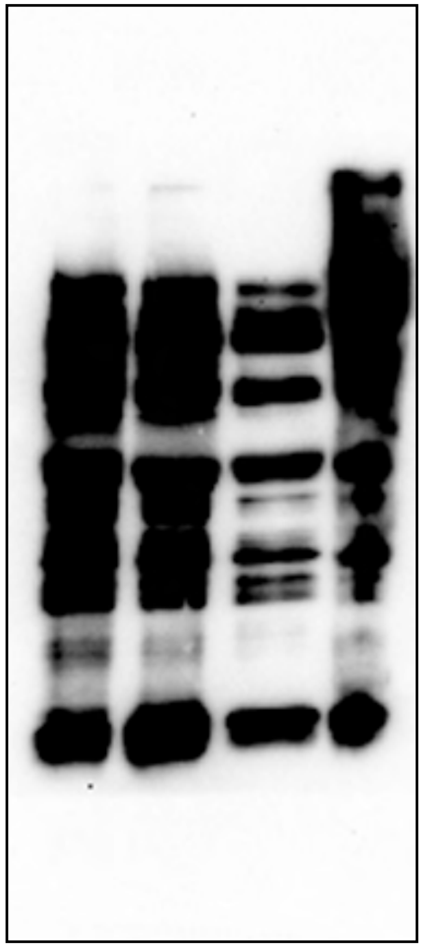

FLAG (PIM1)

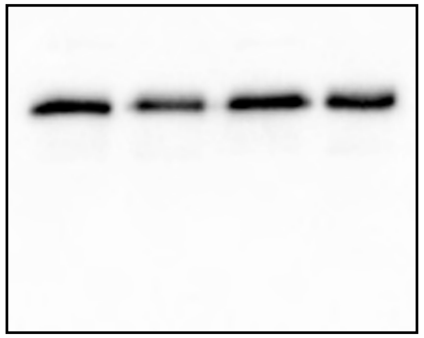

HSP90

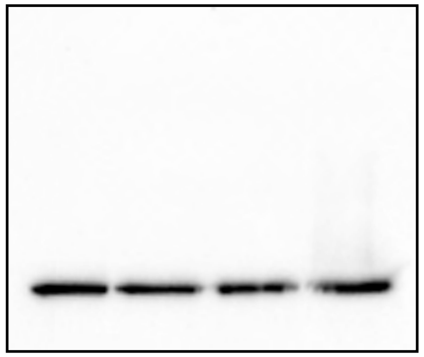

ThioP - AR IP

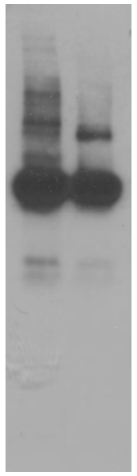

AR - AR IP

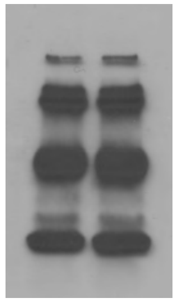

Flag and tubulin - input

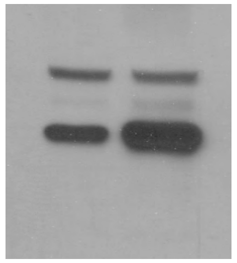

Figure 2

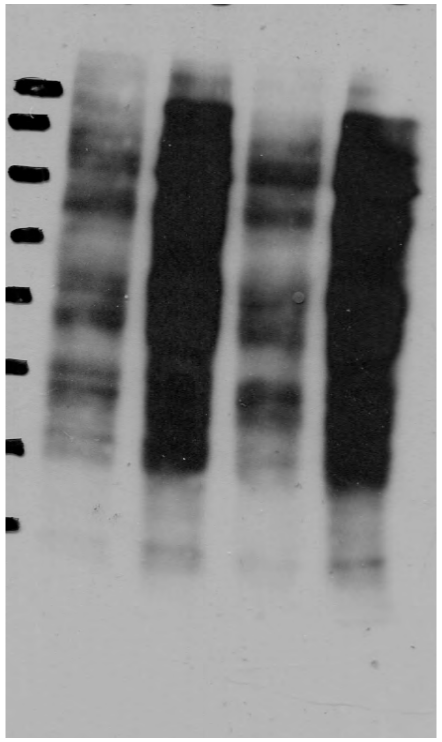

Figure 3 - A-D

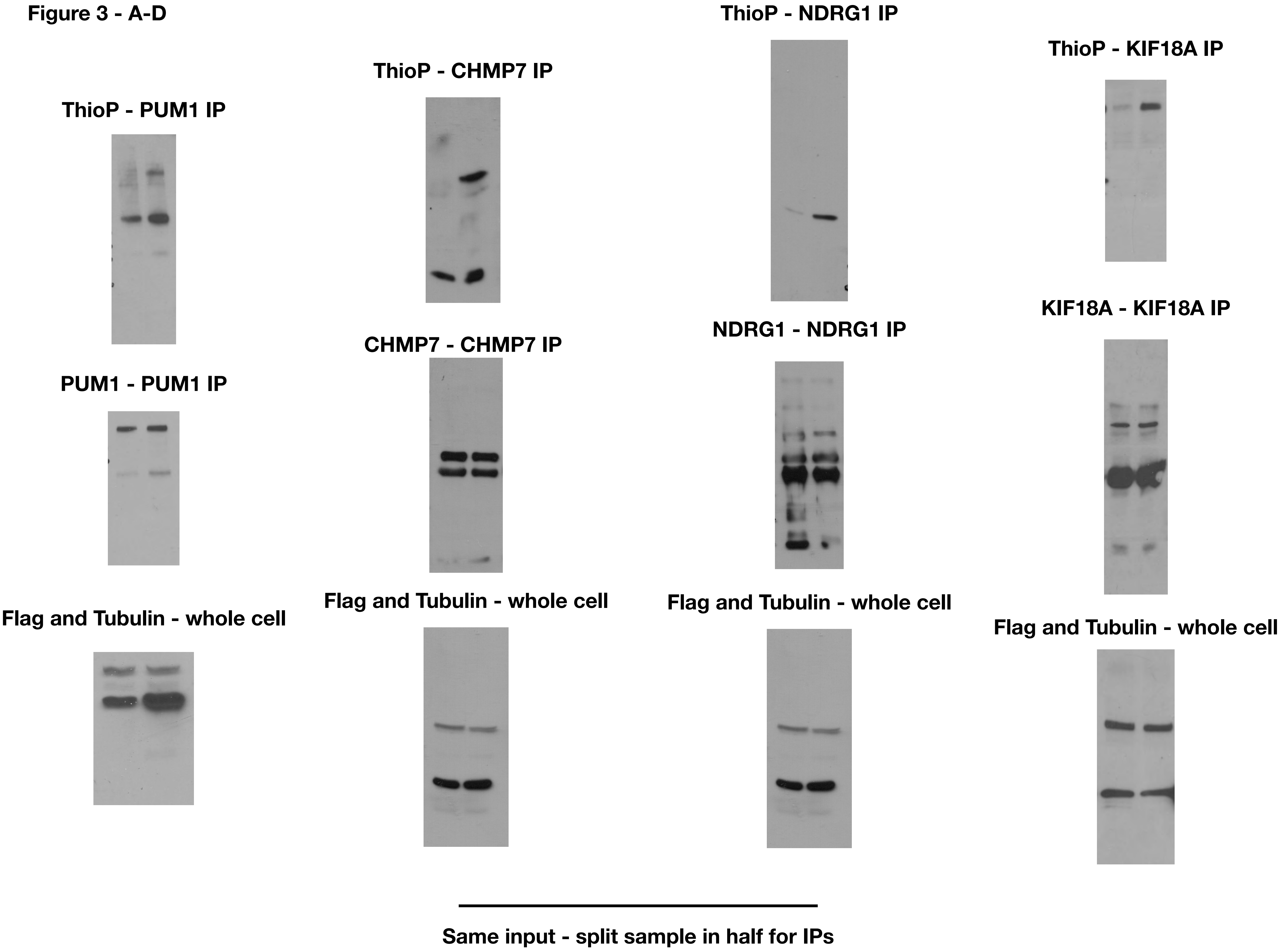
ThioP - PUM1 IP
PUM1 - PUM1 IP
Flag and Tubulin - whole cell
ThioP - CHMP7 IP
CHMP7 - CHMP7 IP
Flag and Tubulin - whole cell
ThioP - NDRG1 IP
NDRG1 - NDRG1 IP
Flag and Tubulin - whole cell
ThioP - KIF18A IP
KIF18A - KIF18A IP
Flag and Tubulin - whole cell
Same input - split sample in half for IPs

Figure 3 - E-H

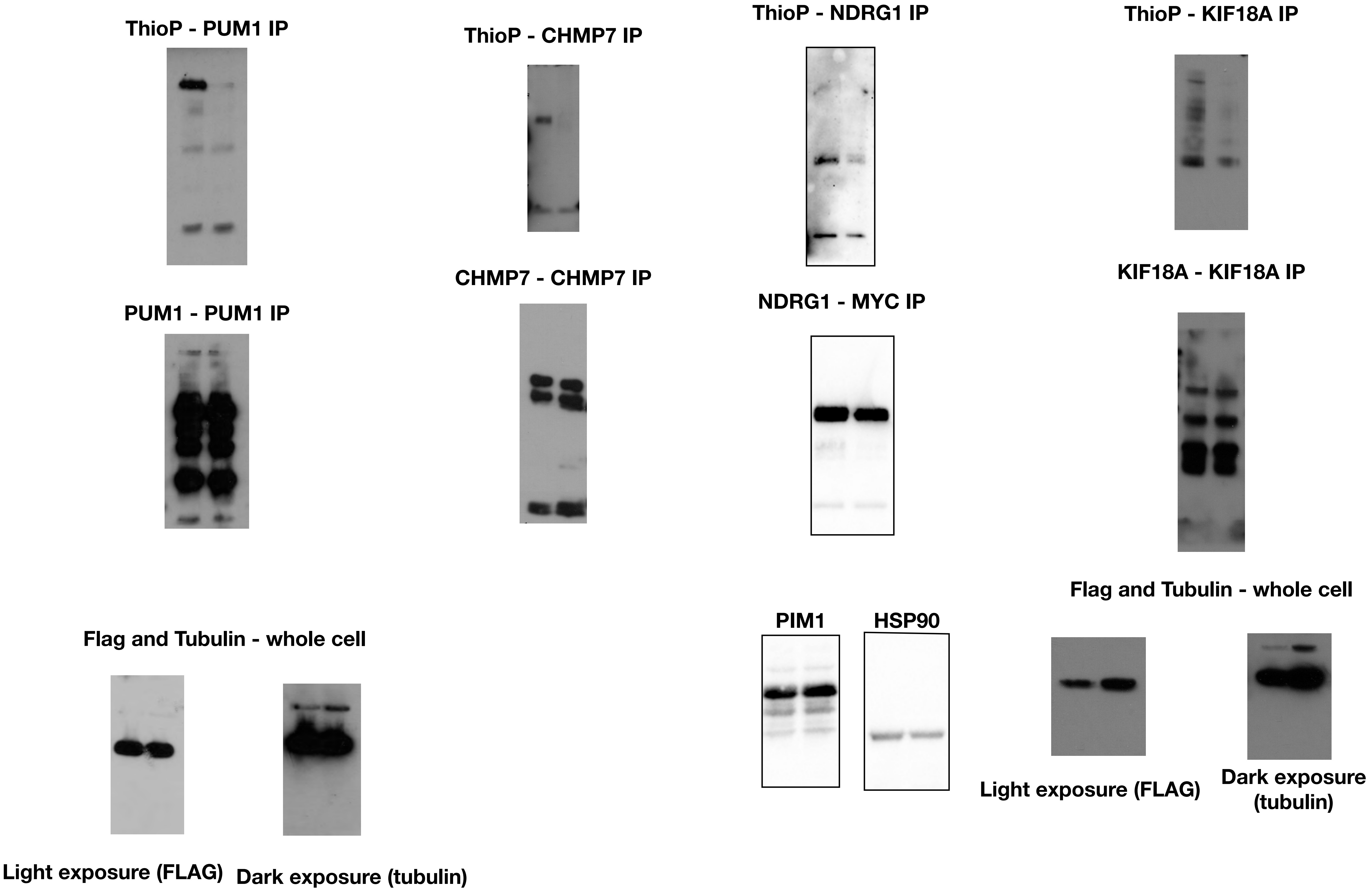
ThioP - PUM1 IP
ThioP - CHMP7 IP
ThioP - NDRG1 IP
ThioP - KIF18A IP
PUM1 - PUM1 IP
CHMP7 - CHMP7 IP
NDRG1 - MYC IP
KIF18A - KIF18A IP
Flag and Tubulin - whole cell
Light exposure (FLAG)
Dark exposure (tubulin)
PIM1
HSP90
Flag and Tubulin - whole cell
Light exposure (FLAG)
Dark exposure (tubulin)

Figure 4

NDRG1 S330

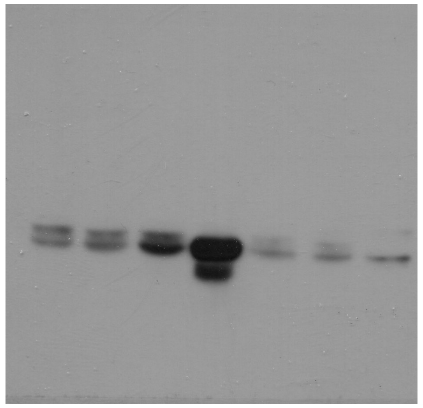

Total NDRG1

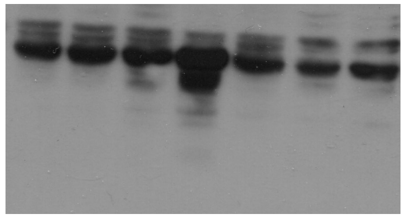

AR

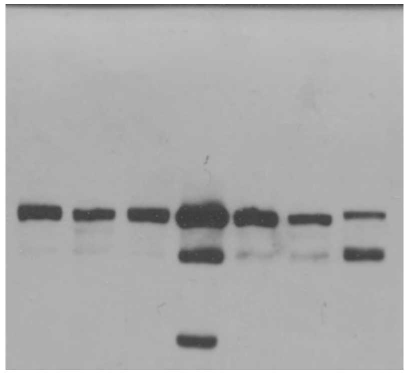

PIM1

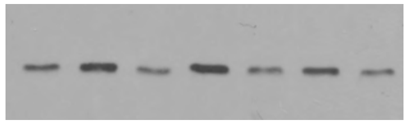

HSP90

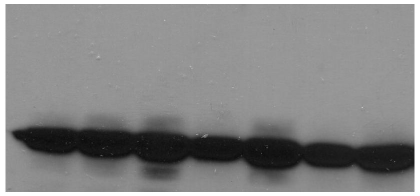

Figure 5 A-C

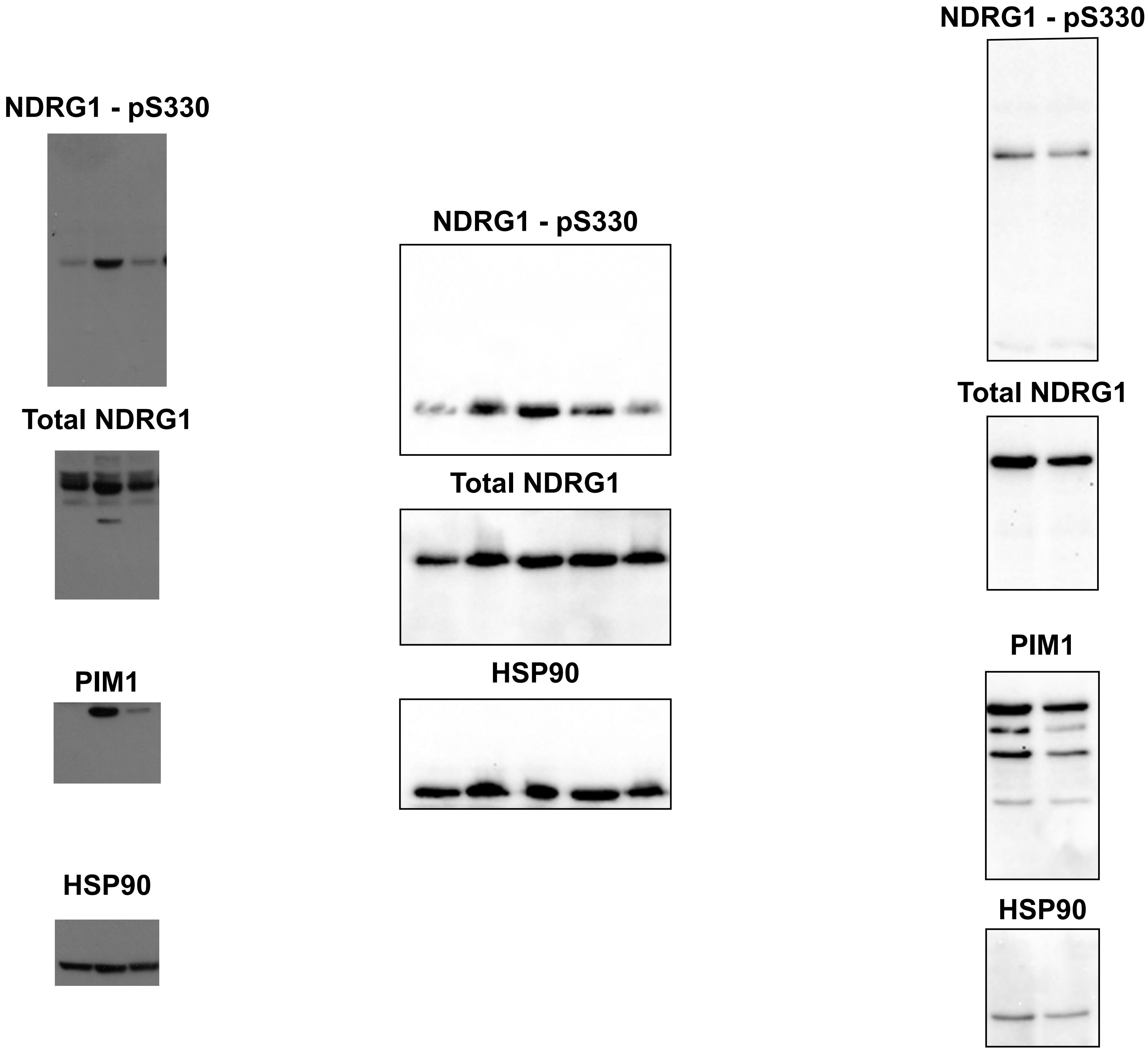
NDRG1 - pS330
Total NDRG1
PIM1
HSP90
NDRG1 - pS330
Total NDRG1
HSP90
NDRG1 - pS330
Total NDRG1
PIM1
HSP90

Figure 5 D, F

NDRG1

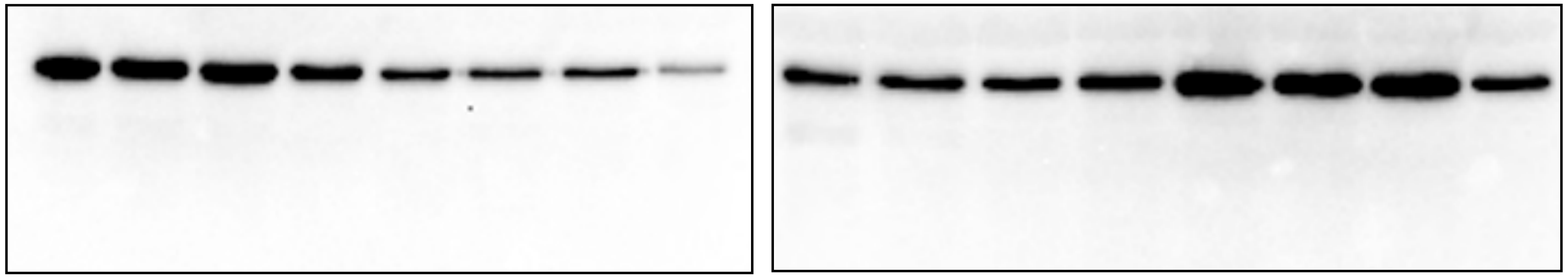

HSP90

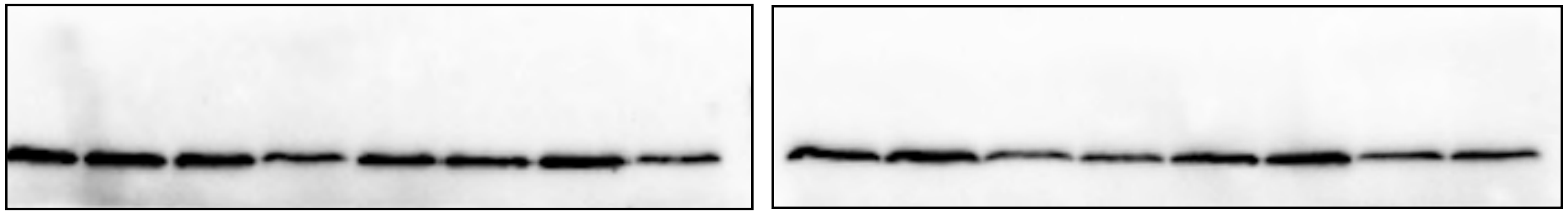

NDRG1

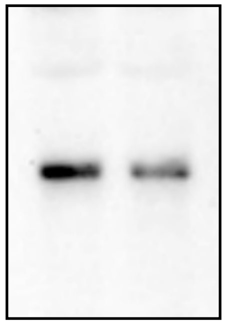

PIM1

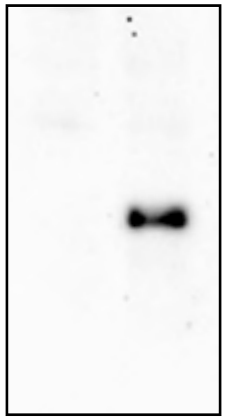

HSP90

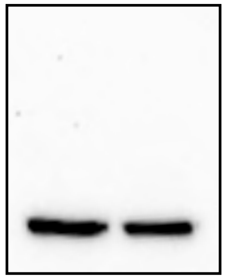

Figure 6A

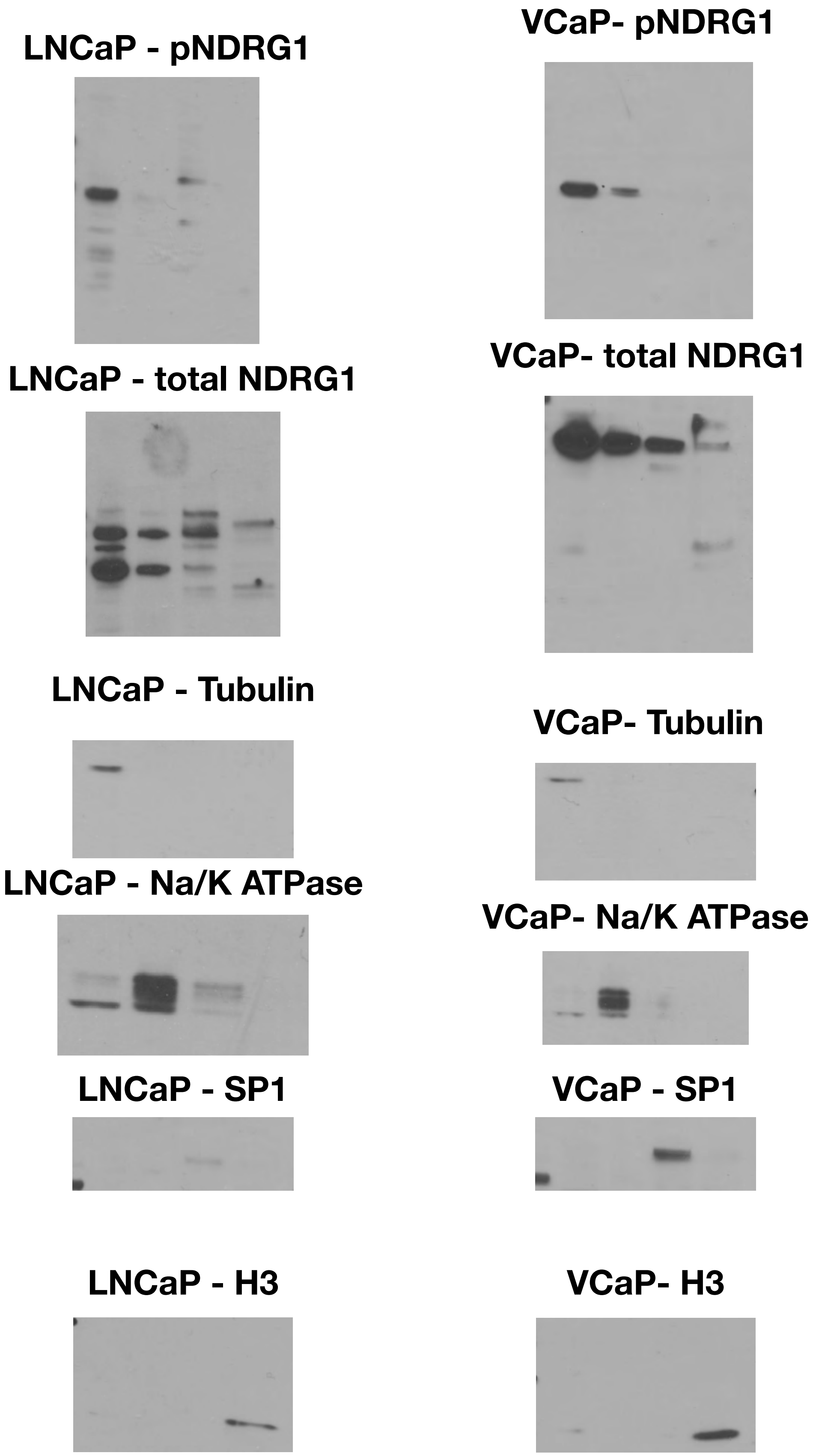
LNCaP - pNDRG1
VCaP- pNDRG1
LNCaP - total NDRG1
VCaP- total NDRG1
LNCaP - Tubulin
VCaP- Tubulin
LNCaP - Na/K ATPase
VCaP- Na/K ATPase
LNCaP - SP1
VCaP - SP1
LNCaP - H3
VCaP- H3

Figure 6B-C

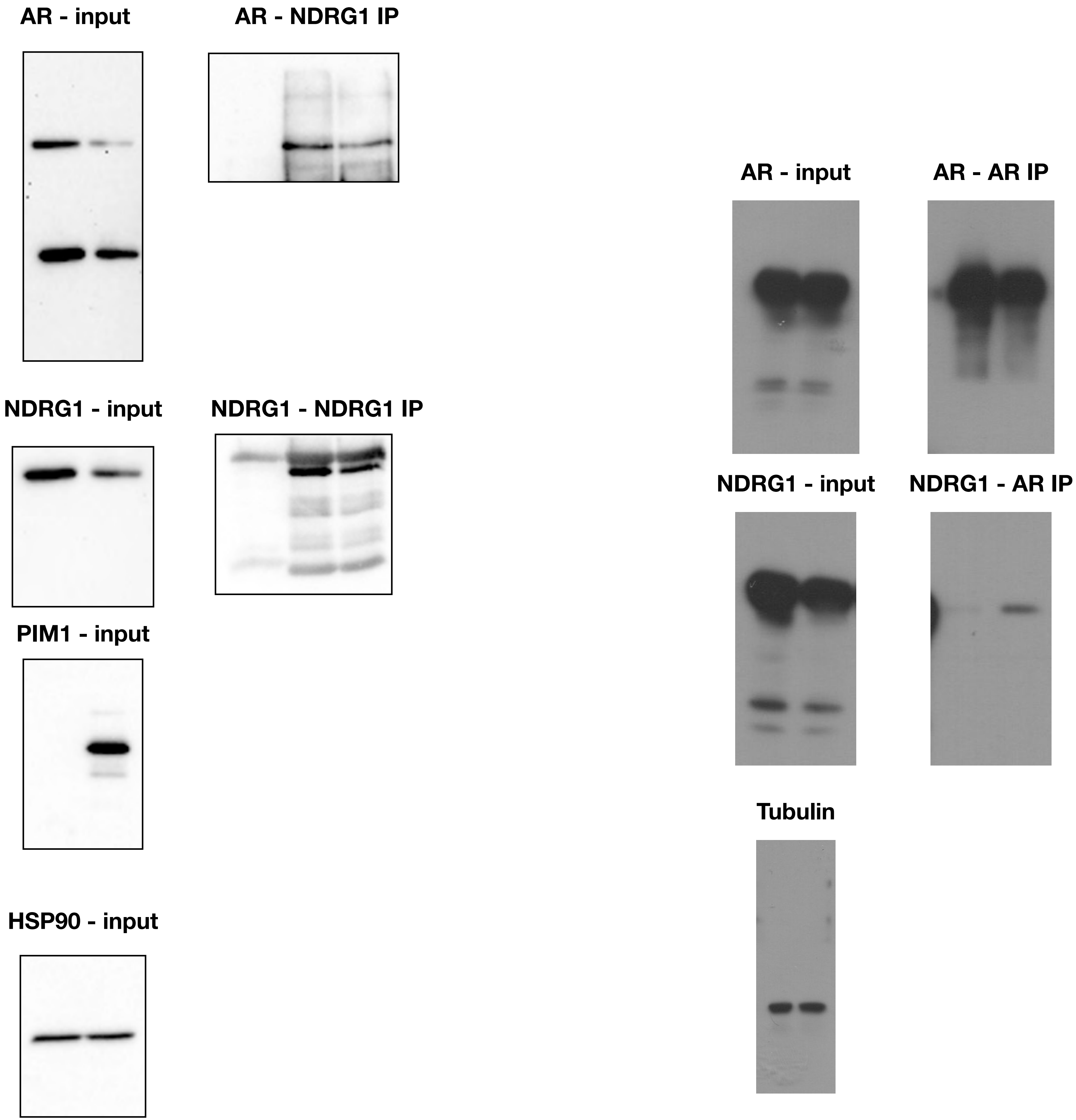
AR - input
AR - NDRG1 IP
NDRG1 - input
NDRG1 - NDRG1 IP
PIM1 - input
HSP90 - input
AR - input
AR - AR IP
NDRG1 - input
NDRG1 - AR IP
Tubulin

Supplementary Table 1. PIM1 Gatekeeper Residue Alignment

**PIM1 Residue**							**120**					
***H.s.* PIM1**	S	H	F	V	L	I	**L**	E	R	P	E	P
H.s. AMPK	T	D	F	F	M	V	**M**	E	Y	V	S	G
H.s. CDK2	N	K	L	Y	L	V	**F**	E	F	L	H	Q
M.m. Jnk1	Q	D	V	Y	I	V	**M**	E	L	M	D	A
M.m. Pka ca	S	N	L	Y	M	V	**M**	E	Y	V	P	G
S.c. CDC28	H	K	L	Y	L	V	**F**	E	F	L	D	L
S.c. CLA4	E	E	L	W	V	I	**M**	E	Y	M	E	G

Supplementary Table 1. PIM1 gatekeeper residue alignment with kinases that analog-sensitive mutants have been generated for references 22,28,69-72.

Supplementary **Table 2. PIM1 Aggregate Substrate Identification**

UniPROT Accession ID	Protein	Identified Phosphosite	Peptides Identified (Exp.1)	Peptides Identified (Exp. 2)	Validated	Function
Q14671	PUM1	T112	32	27	Yes	RNA-binding protein translation control
O75376	NCOR1	S1450	6	6	No	transcriptional regulation/ co-repressor
Q7Z460	CLASP1	S647	6	2	No	microtubule binding protein /stabilizes microtubules
O14531	DPYSL4	S537	4	1	No	semaphorin signaling / cytoskeleton remodeling
P05204	HMGN2	S29	4	4	No	alters interaction between DNA and histone octamer
P07195	LDHB	T302	4	4	No	synthesizes (S)-lactate from pyruvate
Q8WUX9	CHPM7	T408	4	8	Yes	promotes nuclear envelope sealing/ spindle disassembly
O14979	hnRNPDL	T62	2	4	No	DNA/RNA binding protein: transcriptional repressor
Q92597	NDRG1	S330	2	9	Yes	tumor suppressor/ cell trafficking
P55199	ELL	T345	1	1	No	RNA pol II elongation factor
Q02878	RPL6	T92	1	2	No	component of the large 60s ribosomal subunit
Q13428	TCOF1	S1350	1	1	No	regulator of RNA polymerase I
Q14207	NPAT	T115	1	1	No	transcriptional activator / cell cycle progression
Q5QP82	DCAF10	T348	1	1	No	substrate receptor ubiquitin-protein ligase
Q8NI77	KIF18A	T834	1	2	Yes	microtubule-depolymerizer /chromosome congression
P63104	YWHAZ	S64	4	-	No	adapter protein/ regulates signaling
Q13442	PDAP1	S19	2	-	No	enhances PDGFA-stimulated cell growth
Q14676	MDC1	S1113	-	2	No	cell cycle arrest in response to DNA damage
Q7Z628	NET1	S21	-	2	No	GEF for RhoA GTPase.
P59672	ANKS1	S887	1	-	No	regulates EPHA8 receptor signaling to control cell migration
Q96F86	EDC3	S161	2	-	No	mRNA degradation/ promotes mRNA de-capping
Q9H0H5	RACGAP1	T249	-	6	No	myosin contractile ring formation during cytokinesis
P02686	MBP	T229	1	-	No	myelin membrane formation and stabilization
Q99959	PKP2	T320	-	1	No	adherence junction maintenance
P09651	hnRNPA1	S199	7	-	No	mRNA transport from nucleus to cytoplasm

Supplementary Table 2. PIM1 substrates identified from LNCaP cells. UniProt accession ID, phosphorylation site, peptide count from two independent experiments, validation status, and function are listed.

Supplementary Table 3. PIM1 Substrate Phosphosite Alignment (All Substrates)

Protein	Phosphosite	-7	-6	-5	-4	-3	-2	-1	0	1	2	3
PUM1	T112	N	S	K	H	R	W	P	S	G	D	N
NCOR1	S1450	T	V	R	S	R	H	T	S	V	V	S
CLASP1	S647	I	R	T	R	R	Q	S	S	G	S	A
DPYSL4	S537	P	V	R	N	L	H	Q	S	G	F	S
HMGN2	S29	Q	R	R	S	A	R	L	S	A	K	P
LDHB	T302	I	L	N	A	R	G	L	T	S	V	I
CHMP7	T408	N	P	R	N	R	H	F	T	N	S	V
hnRNPDL	T62	R	R	A	Q	R	H	V	T	A	Q	Q
NDRG1	S330	L	M	R	S	R	T	A	S	G	S	S
ELL	T345	K	P	R	I	S	H	F	T	Q	R	A
RPL6	T92	K	K	E	K	V	L	A	T	V	T	K
TCOF1	S1350	E	S	R	K	R	K	L	S	G	D	Q
NPAT	T115	S	Q	R	A	R	T	R	T	G	I	A
DCAF10	T348	I	L	R	A	R	R	T	T	S	S	S
KIF18A	T834	A	K	R	K	R	K	L	T	S	S	T
YWHAZ*	S64	S	S	W	R	V	V	S	S	I	E	Q
PDAP1*	S19	G	R	A	R	Q	Y	T	S	P	E	E
MDC1*	S1113	K	I	R	T	R	K	S	S	R	M	T
NET1*	S21	R	R	R	S	R	R	A	S	G	L	S
ANKS1*	S887	T	G	R	R	R	H	D	S	L	H	D
EDC3*	S161	S	F	R	R	R	H	N	S	W	S	S
RACGAP1*	T249	W	T	R	S	R	R	K	T	G	T	L
MBP*	T229	K	N	I	V	T	P	R	T	P	P	S
PKP2*	T320	S	G	R	R	A	H	L	T	V	G	Q
hnRNPA1*	S199	S	Q	R	G	R	S	G	S	G	N	F

Highlighted residues represent optimal, secondary, or tertiary residues based on **Table 2.** Red, basic residues. Green, hydrophobic residues. Blue, neutral polar residues. *** means substrate only identified in one replicate.**

Supplementary Table 3. PIM1 substrate phosphorylation site alignment.

Supplementary Table 4. **qPCR Primer List from Study**

**RPL6**	
Forward Primer	ATTCCCGATCTGCCATGTATTC
Reverse Primer	TACCGCCGTTCTTGTCACC
**ELL**	
Forward Primer	GATAGGAGCTACGGGCTGTC
Reverse Primer	TTCCTTGAAATCGGATAGATGGC
**HNRNPDL**	
Forward Primer	TCCGCTCCCGCTACTTTAG
Reverse Primer	CCTGCGCCCTCCCTTTATAG
**TCOF1**	
Forward Primer	CGGGAGCTACTTCCCCTGAT
Reverse Primer	CAGAAGGGTTACGGGCTGAG
**LDHB**	
Forward Primer	TGGTATGGCGTGTGCTATCAG
Reverse Primer	TTGGCGGTCACAGAATAATCTTT
**DCAF10**	
Forward Primer	GGACAATTTTCGCACCATGAC
Reverse Primer	ACAGCCGGTTATCAAGAAATCTG
**KIF10A**	
Forward Primer	TGCTGGGAAGACCCACACTAT
Reverse Primer	GCTGGTGTAAAGTAAGTCCATGA
**CHMP7**	
Forward Primer	AAGCCTCTCAAGTGGACTCTT
Reverse Primer	ACAGACGATACACCTCCTCAG
**NCOR1**	
Forward Primer	ACACCGCAGTATTGTCCAAAT
Reverse Primer	CACCTGGTTTGTCTTGATGTTCT
**PUM1**	
Forward Primer	ATGAGCGTTGCATGTGTCTTG
Reverse Primer	GTAGTCCACCATAGCGTCGTC
**CLASP1**	
Forward Primer	CTGTCTCTGCCTTATAGCAACAC
Reverse Primer	CATCTCGAACCTGGCTGTTTG
**DPYSL4**	
Forward Primer	CGTGAATGACGACCAGTCCTT
Reverse Primer	ACGATGAGGTTTTCTCCGATTTG
**NPAT**	
Forward Primer	AGAGCCCGAACGAGAACTG
Reverse Primer	GGTAAAGTGAGCAACTCTGCAC
**ELL**	
Forward Primer	GATAGGAGCTACGGGCTGTC
Reverse Primer	TTCCTTGAAATCGGATAGATGGC
**NDRG1**	
Forward Primer	CTCCTGCAAGAGTTTGATGTCC
Reverse Primer	TCATGCCGATGTCATGGTAGG
**PSA**	
Forward Primer	GTGTGTGGACCTCCATGTTATT
Reverse Primer	CCACTCACCTTTCCCCTCAAG
**Nkx3.1**	
Forward Primer	CCCACACTCAGGTGATCGAG
Reverse Primer	GAGCTGCTTTCGCTTAGTCTT
