# Supplementary material for: Identification of PIM1 substrates reveals a role for NDRG1 phosphorylation in prostate cancer cellular migration and invasion
Source: Commun Biol. 2021 Jan 4;4:36. doi: 10.1038/s42003-020-01528-6 (PMC7782530; doi:10.1038/s42003-020-01528-6)
Supplement: Supplementary file 2 — Description of Additional Supplementary Files [file 42003_2020_1528_MOESM2_ESM.pdf]

Description of Additional Supplementary Files

File Name: Supplementary Data 1
Description: PIM1 substrate phosphorylation in metastatic versus primary prostate cancer from Drake *el al.* [43]

File Name: Supplementary Data 2
Description: Prostate tumor microarray sample data, scoring and statistical analysis.

File Name: Supplementary Data 3
Description: Source data for Fig. 5E, 6E, 6F.
