# Supplementary material for: Identification of PIM1 substrates reveals a role for NDRG1 phosphorylation in prostate cancer cellular migration and invasion
Source: Commun Biol. 2021 Jan 4;4:36. doi: 10.1038/s42003-020-01528-6 (PMC7782530; doi:10.1038/s42003-020-01528-6)
Supplement: Supplementary file 6 — Reporting Summary [file 42003_2020_1528_MOESM6_ESM.pdf]

nature research

Corresponding author(s): Michael Garabedian & Susan Logan

Last updated by author(s): 11/18/2020

Reporting Summary

Nature Research wishes to improve the reproducibility of the work that we publish. This form provides structure for consistency and transparency in reporting. For further information on Nature Research policies, see Authors & Referees and the Editorial Policy Checklist.

Statistics

For all statistical analyses, confirm that the following items are present in the figure legend, table legend, main text, or Methods section.

n/a	Confirmed	
☐	☒	The exact sample size (*n*) for each experimental group/condition, given as a discrete number and unit of measurement
☐	☒	A statement on whether measurements were taken from distinct samples or whether the same sample was measured repeatedly
☐	☒	The statistical test(s) used AND whether they are one- or two-sided<br>*Only common tests should be described solely by name; describe more complex techniques in the Methods section.*
☒	☐	A description of all covariates tested
☐	☒	A description of any assumptions or corrections, such as tests of normality and adjustment for multiple comparisons
☐	☒	A full description of the statistical parameters including central tendency (e.g. means) or other basic estimates (e.g. regression coefficient) AND variation (e.g. standard deviation) or associated estimates of uncertainty (e.g. confidence intervals)
☐	☒	For null hypothesis testing, the test statistic (e.g. *F*, *t*, *r*) with confidence intervals, effect sizes, degrees of freedom and *P* value noted<br>*Give P values as exact values whenever suitable.*
☒	☐	For Bayesian analysis, information on the choice of priors and Markov chain Monte Carlo settings
☒	☐	For hierarchical and complex designs, identification of the appropriate level for tests and full reporting of outcomes
☒	☐	Estimates of effect sizes (e.g. Cohen's *d*, Pearson's *r*), indicating how they were calculated

Our web collection on statistics for biologists contains articles on many of the points above.

Software and code

Policy information about availability of computer code

Data collection	*Provide a description of all commercial, open source and custom code used to collect the data in this study, specifying the version used OR state that no software was used.*
Data analysis	Statistical testing was completed using Prism 7 for Mac OS X, Version 7.0e.

For manuscripts utilizing custom algorithms or software that are central to the research but not yet described in published literature, software must be made available to editors/reviewers. We strongly encourage code deposition in a community repository (e.g. GitHub). See the Nature Research guidelines for submitting code & software for further information.

Data

Policy information about availability of data

All manuscripts must include a data availability statement. This statement should provide the following information, where applicable:

- Accession codes, unique identifiers, or web links for publicly available datasets
- A list of figures that have associated raw data
- A description of any restrictions on data availability

The authors declare that all data supporting the findings of this study are available within the paper and its supplementary information files.

Field-specific reporting

Please select the one below that is the best fit for your research. If you are not sure, read the appropriate sections before making your selection.

☒ Life sciences ☐ Behavioural & social sciences ☐ Ecological, evolutionary & environmental sciences

Life sciences study design

All studies must disclose on these points even when the disclosure is negative.

Sample size	*Describe how sample size was determined, detailing any statistical methods used to predetermine sample size OR if no sample-size calculation was performed, describe how sample sizes were chosen and provide a rationale for why these sample sizes are sufficient.*
Data exclusions	No data were excluded from the analyses.
Replication	All experiments were reproduced 2-3 times and had high reproducibility.
Randomization	Samples were randomly allocated into experimental groups.
Blinding	Blinding was not possible due to labeling of experimental materials, but is not necessary since the methods used are objective quantifications, including absorbance read by spectrophotometer and measurement of distances.

Reporting for specific materials, systems and methods

We require information from authors about some types of materials, experimental systems and methods used in many studies. Here, indicate whether each material, system or method listed is relevant to your study. If you are not sure if a list item applies to your research, read the appropriate section before selecting a response.

Materials & experimental systems

n/a	Involved in the study	
☐	☒	Antibodies
☐	☒	Eukaryotic cell lines
☒	☐	Palaeontology
☒	☐	Animals and other organisms
☒	☐	Human research participants
☒	☐	Clinical data

Methods

n/a	Involved in the study	
☒	☐	ChIP-seq
☒	☐	Flow cytometry
☒	☐	MRI-based neuroimaging

Antibodies

Antibodies used	The antibodies used were as follows: PIM1 (sc-13513; Santa Cruz Biotechnology), NDRG1 (HPA006881, Sigma), NDRG1 pS330 (ab124713, Abcam), AR (sc-7305, Santa Cruz Biotechnology), HSP90 (610418, BD Biosciences), FLAG (F3165, Sigma), α-Tubulin (ab7291, Abcam), CHMP7 (HPA036119, Sigma), Thiophosphate ester antibody [51-8] (ab92570, Abcam), KIF18A (C-19, Santa Cruz Biotechnology), PUM1 (ab92545, Abcam), NaK+ ATPase (EP1845Y, Abcam), SP1 (PA5-29165, Thermofisher), Histone H3 (ab1791, Abcam).
Validation	All antibodies were used for applications which are validated on the manufacturer's website. Additionally, the manuscript includes knockdown and overexpression experiments for NDRG1 and PIM1 which further validate the antibodies.

Eukaryotic cell lines

Policy information about cell lines

Cell line source(s)	Cell lines used in this study were purchased from ATCC, with the exception of LNCaP-9566, LNCaP-abl67, and LAPC4 cells, which were gifts from Dr. J. Luo (Johns Hopkins University), Dr. Z. Culig (Innsbruck Medical University, Austria), and Dr. R. Reiter (University of California, Los Angeles) respectively. C4-2 cells were purchased from Characterized Cell Line Core Facility at MD Anderson Cancer Center (Houston, TX).
Authentication	Both LNCaP-95 and LNCaP-abl cells have been authenticated by short tandem repeat profiling.
Mycoplasma contamination	Cell lines tested negative for mycoplasma testing.
Commonly misidentified lines (See ICLAC register)	N/A
